# Association between weight-adjusted-waist index and chronic kidney disease: a cross-sectional study

**DOI:** 10.1186/s12882-023-03316-w

**Published:** 2023-09-11

**Authors:** Xiaowan Li, Lanyu Wang, Hongyi Zhou, Hongyang Xu

**Affiliations:** 1 Department of Critical Care Medicine, The Affiliated Wuxi People’s Hospital of Nanjing Medical University, Wuxi People’s Hospital, Wuxi Medical Center, Nanjing Medical University, Nanjing, China; 2 Department of Urology, The Affiliated Wuxi People’s Hospital of Nanjing Medical University, Wuxi People’s Hospital, Wuxi Medical Center, Nanjing Medical University, Nanjing, China

**Keywords:** Weight-adjusted-waist index, Chronic kidney disease, Albuminuria, Estimated glomerular filtration rate, Cross-sectional study

## Abstract

**Aims:**

We aimed to investigate the potential association between weight-adjusted-waist index (WWI) and chronic kidney disease (CKD).

**Design and methods:**

This research examined data collected from the National Health and Nutrition Examination Survey (NHANES) spanning from 1999 to 2020. CKD was defined as the low estimated glomerular filtration rate (eGFR) or the existence of albuminuria (urinary albumin-to-creatinine ratio (ACR) ≥ 30mg/g). Low-eGFR was described as eGFR < 60 mL/min/1.73m^2^. The associations between WWI with CKD, albuminuria, and low-eGFR were examined using generalized additive models and weighted multivariable logistic regression models. We also analyzed the associations of other obesity indicators with CKD, albuminuria, and low-eGFR, including body mass index (BMI), waist-to-height ratio (WHtR), waist circumference(WC), height, and weight. The receiver operating characteristic (ROC) curves were used to assess and compare their diagnostic abilities.

**Results:**

Males made up 48.26% of the total 40,421 individuals that were recruited. The prevalences of CKD, albuminuria, and low-eGFR were 16.71%, 10.97%, and 7.63%, respectively. WWI was found to be positively linked with CKD (OR = 1.42; 95% CI: 1.26, 1.60). A nonlinear connection between WWI and CKD was found using smooth curve fitting. Additionally, a higher prevalence of albuminuria is linked to a higher level of WWI (OR = 1.60; 95% CI: 1.40, 1.82). Different stratifications did not substantially influence the connection between WWI and CKD, albuminuria, and low-eGFR, according to subgroup analysis and interaction tests. We observed higher height was related to higher low-eGFR prevalence (OR = 1.05; 95% CI: 1.03, 1.06). ROC analysis revealed that WWI had the best discrimination and accuracy for predicting CKD and albuminuria compared to other obesity indicators (BMI, WHTR, WC, height and weight). In addition, height had the highest area under the curve (AUC) value for predicting low-eGFR.

**Conclusion:**

WWI is the best obesity indicator to predict CKD and albuminuria compared to other obesity indicators (BMI, WHTR, WC, height, and weight). WWI and CKD and albuminuria were found to be positively correlated. Furthermore, height had the strongest ability to predict low-eGFR. Therefore, the importance of WWI and height in assessing kidney health in US adults should be emphasized.

**Supplementary Information:**

The online version contains supplementary material available at 10.1186/s12882-023-03316-w.

## Introduction

A major contributor to morbidity and death globally is chronic kidney disease (CKD), which is characterized by structural or functional abnormalities of the kidneys brought on by a number of factors. CKD was defined as the low estimated glomerular filtration rate (eGFR) or the existence of albuminuria. Low-eGFR was described as eGFR < 60 mL/min/1.73m^2^, while albuminuria was defined as urinary albumin-to-creatinine ratio (ACR) ≥ 30 mg/g [1]. There were 697.5 million people with CKD worldwide in 2017, which led to 1.2 million fatalities and 35.8 million disability-adjusted life years (DALYs) [2]. As a result, healthcare practice should place high importance on kidney health. CKD is at risk of being caused by cardiovascular disease, hypertension, diabetes, and obesity. An increasingly prominent risk factor that is modifiable is obesity [3]. Exploring possible modifiable risk factors for CKD is therefore becoming more and more crucial and may present fresh opportunities for prevention.

Obesity has become a significant problem for public health on a global scale. Both domestically and internationally, obesity has increased in prevalence over the last few decades [4]. By 2030, it is anticipated that nearly half of US adults would be obese [5]. In 2018, Park et al. proposed weight-adjusted-waist index (WWI) as a new obesity metric [6]. It is an anthropometric indicator of central obesity that takes into consideration both muscle and fat mass and is derived from the formula waist circumference (WC) divided by the square root of body weight [7, 8]. The prevalences of newly diagnosed hypertension, diabetes, cardiovascular disease, and even all-cause and cardiovascular death have all been shown to be strongly linked with WWI [6, 9, 10]. However, previous literature examining WWI and kidney function is sparse, with only Zheng et al. demonstrating a positive connection between WWI and albuminuria [11]. As far as we are aware, no research has examined the link between WWI and CKD.

Consequently, using information from the National Health and Nutrition Examination Survey (NHANES), this study intends to examine the connection between WWI and CKD.

## Materials and methods

### Survey description

NHANES provided cross-sectional data. The National Center for Health Statistics (NCHS) conducts NHANES surveys to gauge the nutritional and physical health of the non-institutionalized population in the United States [12]. The NHANES survey data is being updated while it is still in its 2-year repeat cycle. The stratified multi-stage probabilistic strategy utilized in the NHANES study design results in a relatively large representation among the enrolled participants. The NCHS research ethics review committee gave its approval to all NHANES survey procedures, and each study participant signed informed consent. Please visit the official NHANES website for further details on the planning and execution.

### Study population

**Fig. 1:**
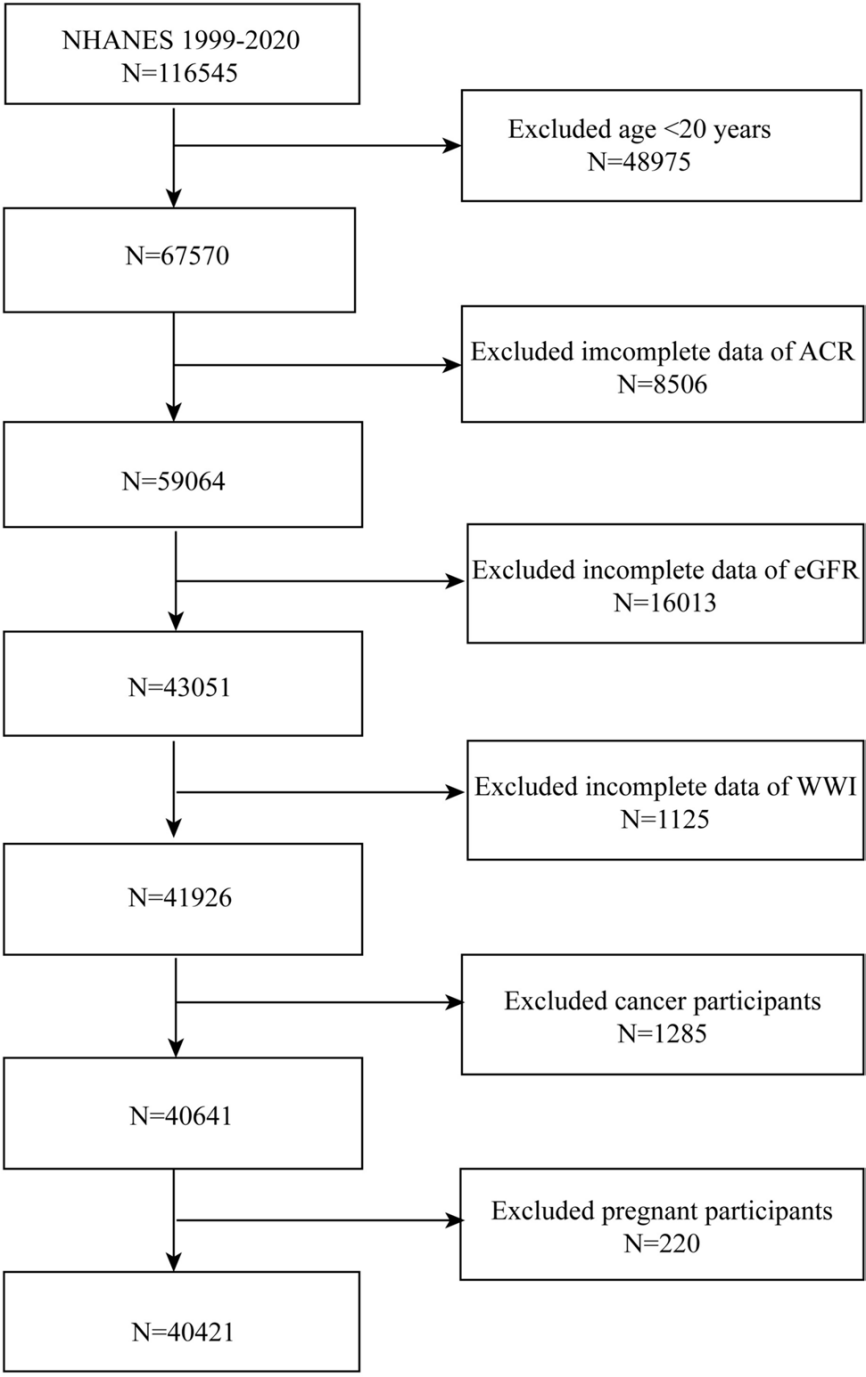
Flowchart of the sample selection from NHANES 1999–2020

We drew participants for our study from the NHANES 1999–2020. After removing patients who were < 20 years old (*n* = 48,975), having cancer (*n* = 1,285) and pregnant (*n* = 220), missing ACR (*n* = 8,506), eGFR (*n* = 16,013), and WWI (*n* = 1,125) from the study, we were left with 40,421 eligible subjects (Fig. 1).

### Definition of WWI and CKD

WWI was regarded as an exposure variable. WWI is an anthropometric index that estimates the degree of obesity by combining data on WC and body weight. The WC of each participant was divided by the square root of their body weight, and the result was rounded to two decimal places. Higher WWI scores are indicative of higher levels of obesity. Trained medical technicians gathered anthropometric information on WC and body weight at the mobile examination center (MEC). The weight was measured with a digital weight scale to the nearest 0.1 kg. Each subject was weighed while clad in the MEC examination attire, standing in the center of the digital scale, his hands by his sides, and his eyes focused straight ahead. A retractable steel tape measure was used to compute WC. The right iliac crest was palpated on both sides, and a horizontal line was then drawn above its most superior lateral border. The next step was to draw a line across the right midaxillary. The intersection of the two lines is where the tape measure is located on the horizontal plane. The measurements will be obtained and rounded to the nearest 0.1 cm when the person's normal expiration ends. To more fully assess the association between WWI and CKD, we similarly evaluated the association of other obesity indicators with renal function, including body mass index (BMI)(BMI = weight (kg)/height^2^ (m)), waist-to-height ratio (WHtR)(WHtR = WC (cm)/height (cm)), WC, height and weight.

Albuminuria or the eGFR of less than 60 mL/min/1.73 m^2^ is required for the diagnosis of CKD [1]. The Chronic Kidney Disease Epidemiology Collaboration (CKD-EPI) equation for standardized creatinine was used to calculate eGFR [13]. Albuminuria was defined as ACR ≥ 30 mg/g. For the sake of discussion, we consider eGFR, low-eGFR, and CKD in this study to represent eGFR(CKD-EPI), low-eGFR(CKD-EPI), and CKD (CKD-EPI)(all calculated by the CKD-EPI equation for standardized creatinine). This research mainly focused on low-eGFR, CKD, and albuminuria as outcome variables. We also assessed the association of different obesity indicators with CKD (EKFC) and low-eGFR (EKFC) in the Supplementary Materials. We calculated eGFR (EKFC) using the European Kidney Function Consortium (EKFC) formula [14]. CKD (EKFC) was diagnosed by albuminuria or eGFR (EKFC) < 60 mL/min/1.73 m^2^.

### Selection of covariates

Our study controlled for several demographic covariates, including sex (male/female), age (year), race (Mexican American/other Hispanic/non-Hispanic White/non-Hispanic Black/other races), and education level (less than high school/high school or general educational development (GED)/above high school/others). In addition, we also included several self-reported daily behaviors and laboratory covariates, such as smoking status (≥ 100 cigarettes lifetime/ < 100 cigarettes lifetime), serum uric acid (mg/dL), total cholesterol (TC) (mg/dL), high-density lipoprotein cholesterol (HDL-C) (mg/dL), low-density lipoprotein cholesterol (LDL-C) (mg/dL), triglycerides (mg/dL) and serum total calcium (mg/dL).

We also included health status differences, such as hypertension and diabetes, as covariates in our analysis. The definition of hypertension used in this study comprises three parts. The first part includes a self-report of hypertension based on the questionnaire item "Ever told you you had hypertension." Measuring mean systolic or mean diastolic blood pressure above 130 or 80 mmHg is part of the second Sect [15]. The third part involves identifying hypertensive participants based on the item "taking hypertension prescription" program. In the case of diabetes, the definition used involved three parts as well. Self-reported diabetes made up the first section, while the usage of insulin or diabetes medications made up the second. The final component entailed identifying patients with diabetes using fasting glucose (mmol/l) ≥ 7.0 and glycohemoglobin or hemoglobin A1c (HbA1c) (%) > 6.5. You may get all the information about these variables on the internet at www.cdc.gov/nchs/nhanes/.

### Statistical analysis

Following the recommendations of the U.S. Centers for Disease Control and Prevention (CDC), the complicated sample design of a multi-stage cluster survey was taken into account in all statistical analyses [16]. The mean and standard deviation of continuous variables were displayed, whilst percentages were used to display categorical variables. Differences across groups of WWI (tertiles) were examined for categorical or continuous variables using weighted t-tests or weighted chi-square tests. The associations between WWI and CKD, albuminuria, and low-eGFR was investigated in Models 1–3 using weighted multivariable regression models. No covariate was adjusted in Model 1 at all. Model 2 adjusted for sex, age, and race. Model 3 was adjusted to account for factors such as sex, age, race, education level, smoking status, serum uric acid, TC, LDL-C, HDL-C, triglycerides, serum total calcium, hypertension, and diabetes status. Model 4 was adjusted for albuminuria, sex, age, race, education level, smoking status, serum uric acid, TC, LDL-C, HDL-Cl, triglycerides, total serum calcium, hypertension, and diabetes status. We also investigated the relationship between WWI and GFR in Models 1,3 and 4. WWI was converted from a continuous variable to a categorical variable (tertiles) for sensitivity analysis to evaluate its robustness. Non-linear problems were addressed using smooth curve fitting and generalized additive models (GAM). When non-linear correlations were seen, the threshold effect was calculated by fitting each interval with a two-segment linear regression model (segmented regression model). The log-likelihood ratio test was used to compare the one-line model (non-segmented) with the two-piecewise linear regression model to see if a threshold exists. A two-step recursive method was used to further identify the breakpoint (K). The connections between WWI with CKD, albuminuria, and low-eGFR was also examined using subgroup analysis utilizing stratified multivariable logistic regression models, stratified by sex, age, smoking status, hypertension, and diabetes. In order to evaluate the heterogeneity of correlations among subgroups, these stratification characteristics were also taken into account as previously mentioned potential impact modifiers. Finally, we analyzed the ability of WWI and other obesity indicators (BMI, WHTR, WC, height and weight) to predict CKD, albuminuria, and low-eGFR by the receiver operating characteristic (ROC) curves and compared areas under the curve (AUC) values. For missing values in categorical variables based on existing data, mode imputation was employed, whereas median imputation was utilized for missing values in continuous variables. R version 4.1.3 and the Empower software package (www.empowerstats.com) was used for all statistical analyses. Statistical significance was set at a two-tailed *p*-value < 0.05.

## Results

### Participants characteristics at baseline

Our analysis included 40,421 participants, with a mean age of 48.75 ± 18.13 years, 48.26% men and 51.74% women. The prevalences of CKD, albuminuria, and low-eGFR were 16.71%, 10.97%, and 7.63%, respectively, with a mean WWI of 10.81 ± 0.93 cm/kg. Among participants in the lowest WWI tertile, 13.97% had CKD and 8.82% had albuminuria. In the middle WWI tertile, 13.55% had CKD and 8.50% had albuminuria. The highest tertile of patients had the highest prevalences of albuminuria (15.58%) and CKD (22.61%). Participants in the higher WWI tertiles had higher prevalences of low-eGFR (Tertile 1: 5.52%; Tertile 2: 6.29%; Tertile 3: 11.08%; *p* < 0.001) (Table 1). The prevalences of CKD (EKFC) and low-eGFR (EKFC) were 18.97% and 10.12%, respectively.

**Table 1:** Baseline characteristics according to WWI tertiles

WWI	Overall	Tertile 1	Tertile 2	Tertile 3	*P-*value
		(5.65–10.38)	(10.38–11.22)	(11.22–15.70)	
N	40,421	13,474	13,472	13,475	
WWI, cm/√kg	10.81 ± 0.93	9.79 ± 0.42	10.80 ± 0.24	11.84 ± 0.48	< 0.001
BMI, kg/m^2^	27.50 ± 6.69	23.35 ± 4.43	27.55 ± 5.74	31.61 ± 6.90	< 0.001
WHTR	0.57 ± 0.10	0.47 ± 0.05	0.56 ± 0.06	0.66 ± 0.08	< 0.001
Height, cm	166.30 ± 10.07	169.39 ± 9.88	166.58 ± 9.75	162.93 ± 9.49	< 0.001
Weight, kg	76.41 ± 20.87	67.54 ± 16.30	77.18 ± 20.13	84.50 ± 22.16	< 0.001
WC, cm	94.01 ± 17.16	79.91 ± 10.41	94.15 ± 12.71	107.98 ± 14.81	< 0.001
Age, years					< 0.001
20–40	15,340 (37.95%)	4705 (34.92%)	5322 (39.50%)	5313 (39.43%)	
41–60	13,075 (32.35%)	4421 (32.81%)	4365 (32.40%)	4289 (31.83%)	
> 60	12,006 (29.70%)	4348 (32.27%)	3785 (28.10%)	3873 (28.74%)	
Sex, *n* (%)					0.533
Male	19,507 (48.26%)	6543 (48.56%)	6511 (48.33%)	6453 (47.89%)	
Female	20,914 (51.74%)	6931 (51.44%)	6961 (51.67%)	7022 (52.11%)	
Race, *n* (%)					0.250
Mexican American	6945 (17.18%)	2293 (17.02%)	2315 (17.18%)	2337 (17.34%)	
Other Hispanic	3470 (8.58%)	1129 (8.38%)	1159 (8.60%)	1182 (8.77%)	
Non-Hispanic White	17,449 (43.17%)	5854 (43.45%)	5778 (42.89%)	5817 (43.17%)	
Non-Hispanic Black	8604 (21.29%)	2942 (21.83%)	2852 (21.17%)	2810 (20.85%)	
Other Races	3953 (9.78%)	1256 (9.32%)	1368 (10.15%)	1329 (9.86%)	
Education level, *n* (%)					0.075
Less than high school	10,581 (26.24%)	3613 (26.86%)	3456 (25.72%)	3512 (26.13%)	
High school or GED	9298 (23.05%)	3131 (23.28%)	3052 (22.71%)	3115 (23.17%)	
Above high school	20,452 (50.71%)	6707 (49.86%)	6929 (51.57%)	6816 (50.70%)	
Others	85 (0.21%)	21 (0.16%)	34 (0.25%)	30 (0.22%)	
Smoking status, *n* (%)					< 0.001
≥ 100 cigarettes lifetime	13,486 (44.24%)	2806 (39.43%)	4843 (44.51%)	5837 (46.73%)	
< 100 cigarettes lifetime	17,000 (55.76%)	4310 (60.57%)	6037 (55.49%)	6653 (53.27%)	
Hypertension, *n* (%)	20,463 (50.73%)	7326 (54.42%)	6356 (47.27%)	6781 (50.50%)	< 0.001
Diabetes, *n* (%)	21,974 (54.73%)	6589 (49.03%)	7138 (53.33%)	8247 (61.88%)	< 0.001
Serum uric acid, mg/dL	5.42 ± 1.55	5.41 ± 1.55	5.48 ± 1.63	5.36 ± 1.49	0.062
TC, mg/dL	183.70 ± 41.64	168.99 ± 35.95	188.51 ± 40.80	193.61 ± 43.68	< 0.001
HDL-C, mg/dL	53.34 ± 15.42	56.07 ± 14.72	53.02 ± 15.87	51.21 ± 15.22	< 0.001
LDL-C, mg/dL	106.90 ± 34.84	96.56 ± 31.14	111.26 ± 34.60	112.85 ± 36.23	< 0.001
Triglyceride, mg/dL	116.06 ± 99.03	84.30 ± 65.43	119.49 ± 99.27	143.96 ± 115.89	< 0.001
Serum total calcium, mg/dL	9.48 ± 0.42	9.47 ± 0.44	9.49 ± 0.41	9.46 ± 0.40	0.064
ACR, mg/g	34.83 ± 259.88	20.61 ± 106.04	25.68 ± 191.42	58.21 ± 392.28	< 0.001
Albuminuria, *n* (%)	4434 (10.97%)	1189 (8.82%)	1145 (8.50%)	2100 (15.58%)	< 0.001
eGFR, mL/min/1.73 m^2^	98.52 ± 28.69	98.04 ± 26.41	99.81 ± 27.69	97.70 ± 31.67	< 0.001
Low-eGFR, *n* (%)	3085 (7.63%)	744 (5.52%)	848 (6.29%)	1493 (11.08%)	< 0.001
CKD, *n* (%)	6755 (16.71%)	1882 (13.97%)	1826 (13.55%)	3047 (22.61%)	< 0.001
eGFR(EKFC), mL/min/1.73 m^2^	88.90 ± 21.71	88.63 ± 19.99	90.12 ± 21.13	87.94 ± 23.78	< 0.001
Low-eGFR(EKFC), *n* (%)	4090 (10.12%)	1111 (8.25%)	1160 (8.61%)	1819 (13.50%)	< 0.001
CKD(EKFC), *n* (%)	7667 (18.97%)	2232 (16.57%)	2107 (15.64%)	3328 (24.70%)	< 0.001

*WWI* Weight-adjusted-waist index, *BMI* body mass index, *WHtR* waist-to-height ratio, *WC* waist circumference, *GED* general educational development, *TC* total cholesterol, *HDL-C* high density lipoprotein-cholesterol, *LDL-C* low-density lipoprotein cholesterol, *ACR* urinary albumin-to-creatinine ratio, *eGFR* urinary albumin-to-creatinine ratio, *CKD* chronic kidney disease, *EKFC* European Kidney Function Consortium

Age, smoking status, hypertension, diabetes, TC, HDL-C, LDL-C, triglycerides, ACR, eGFR, eGFR (EKFC), BMI, WHTR, WC, height and weight all differed significantly between tertiles (all *p* < 0.05). There were no appreciable variations in the tertiles of WWI, though, in terms of sex, race, education level, serum uric acid, or serum total calcium (all *p* > 0.05) (Table 1).

### Association between WWI and CKD

Table 2 shows the associations of WWI and other obesity indicators with CKD. We found positive associations between WWI and other obesity indicators with CKD in both Model 1 and Model 2. In Model 3, WWI, WHTR, and WC were still positively associated with CKD (WWI: OR = 1.42; 95% CI: 1.26, 1.60; WHTR: OR = 7.00; 95% CI: 2.41, 20.36; WC: OR = 1.01; 95% CI: 1.00, 1.02). We also conducted sensitivity analysis by converting WWI and other obesity indicators from continuous variables to categorical variables (tertiles). In Model 3, participants in the highest WWI, WHTR, and WC tertiles had an 87%, 67%, and 39% higher prevalence of CKD than those in the lowest tertiles (WWI: OR = 1.87; 95% CI: 1.42, 2.46; WHTR: OR = 1.67; 95% CI: 1.26, 2.21; WC: OR = 1.39; 95% CI: 1.04, 1.85) (all *p* for trend < 0.05). We also found similar associations between WWI and other obesity indicators with CKD (EKFC) (Supplementary Table S1).

**Table 2:** Associations between WWI and other obesity indicators with CKD, albuminuria, and low-eGFR

**Index**	**Outcome**	**Continuous or categories**	Model 1^c^		Model 2^d^		Model 3^e^	
			OR^a^ (95%CI^b^)	*P-* value	OR (95%CI)	*P-* value	OR (95%CI)	*P-* value
**WWI**	**CKD**	WWI as continuous variable	1.34 (1.30, 1.38)	< 0.0001	1.36 (1.32, 1.40)	< 0.0001	1.42 (1.26, 1.60)	< 0.0001
		Tertile 1	Reference		Reference		Reference	
		Tertile 2	0.97 (0.90, 1.04)	0.3244	1.00 (0.93, 1.07)	0.9571	1.06 (0.79, 1.41)	0.7053
		Tertile 3	1.80 (1.69, 1.92)	< 0.0001	1.88 (1.76, 2.01)	< 0.0001	1.87 (1.42, 2.46)	< 0.0001
		*P* for trend	< 0.0001		< 0.0001		< 0.0001	
	**Albuminuria**	WWI as continuous variable	1.41 (1.36, 1.46)	< 0.0001	1.41 (1.36, 1.46)	< 0.0001	1.60 (1.40, 1.82)	< 0.0001
		Tertile 1	Reference		Reference		Reference	
		Tertile 2	0.96 (0.88, 1.04)	0.3425	0.97 (0.89, 1.05)	0.4295	1.28 (0.92, 1.78)	0.1379
		Tertile 3	1.91 (1.77, 2.06)	< 0.0001	1.92 (1.78, 2.07)	< 0.0001	2.35 (1.73, 3.21)	< 0.0001
		*P* for trend	< 0.0001		< 0.0001		< 0.0001	
	**Low-eGFR**	WWI as continuous variable	1.40 (1.35, 1.46)	< 0.0001	1.46 (1.40, 1.52)	< 0.0001	1.10 (0.95, 1.28)	0.2038
		Tertile 1	Reference		Reference		Reference	
		Tertile 2	1.15 (1.04, 1.27)	0.0072	1.24 (1.12, 1.38)	< 0.0001	0.77 (0.52, 1.13)	0.1756
		Tertile 3	2.13 (1.95, 2.34)	< 0.0001	2.39 (2.17, 2.63)	< 0.0001	1.16 (0.81, 1.66)	0.4184
		*P* for trend	< 0.0001		< 0.0001		0.1607	
**BMI**	**CKD**	BMI as continuous variable	1.01 (1.01, 1.01)	< 0.0001	1.01 (1.01, 1.02)	< 0.0001	1.01 (0.99, 1.02)	0.2558
		Tertile 1	Reference		Reference		Reference	
		Tertile 2	1.04 (0.97, 1.11)	0.2304	1.08 (1.01, 1.15)	0.0247	0.86 (0.66, 1.13)	0.2860
		Tertile 3	1.24 (1.16, 1.32)	< 0.0001	1.30 (1.22, 1.39)	< 0.0001	1.14 (0.87, 1.49)	0.3418
		*P* for trend	< 0.0001		< 0.0001		0.1254	
	**Albuminuria**	BMI as continuous variable	0.99 (0.99, 1.00)	0.9306	1.00 (0.99, 1.01)	0.8757	1.00 (0.98, 1.02)	0.9579
		Tertile 1	Reference		Reference		Reference	
		Tertile 2	0.75 (0.70, 0.81)	< 0.0001	0.76 (0.70, 0.82)	< 0.0001	0.64 (0.48, 0.85)	0.0024
		Tertile 3	0.99 (0.92, 1.07)	0.7818	0.99 (0.93, 1.08)	0.9696	0.91 (0.69, 1.21)	0.5365
		*P* for trend	0.7156		0.5499		0.6806	
	**Low-eGFR**	BMI as continuous variable	1.03 (1.03, 1.04)	< 0.0001	1.04 (1.03, 1.05)	< 0.0001	1.01 (0.99, 1.03)	0.4459
		Tertile 1	Reference		Reference		Reference	
		Tertile 2	2.00 (1.81, 2.22)	< 0.0001	2.26 (2.04, 2.50)	< 0.0001	1.56 (1.08, 2.25)	0.0178
		Tertile 3	2.09 (1.89, 2.31)	< 0.0001	2.42 (2.18, 2.68)	< 0.0001	1.35 (0.93, 1.95)	0.1160
		*P* for trend	< 0.0001		< 0.0001		0.3435	
**WHTR**	**CKD**	WHTR as continuous variable	5.41 (4.22, 6.95)	< 0.0001	6.73 (5.23, 8.67)	< 0.0001	7.00 (2.41, 20.36)	0.0004
		Tertile 1	Reference		Reference		Reference	
		Tertile 2	1.02 (0.95, 1.09)	0.6272	1.05 (0.98, 1.13)	0.1341	1.05 (0.80, 1.40)	0.7122
		Tertile 3	1.55 (1.46, 1.66)	< 0.0001	1.64 (1.54, 1.75)	< 0.0001	1.67 (1.26, 2.21)	0.0004
		*P* for trend	< 0.0001		< 0.0001		< 0.0001	
	**Albuminuria**	WHTR as continuous variable	4.11 (3.05, 5.53)	< 0.0001	4.28 (3.18, 5.76)	< 0.0001	7.19 (2.35, 22.00)	0.0006
		Tertile 1	Reference		Reference		Reference	
		Tertile 2	0.80 (0.74, 0.87)	< 0.0001	0.80 (0.74, 0.87)	< 0.0001	0.97 (0.71, 1.33)	0.8710
		Tertile 3	1.42 (1.32, 1.53)	< 0.0001	1.43 (1.33, 1.54)	< 0.0001	1.78 (1.31, 2.42)	0.0002
		*P* for trend	< 0.0001		< 0.0001		< 0.0001	
	**Low-eGFR**	WHTR as continuous variable	16.54 (11.76, 23.27)	< 0.0001	29.18 (20.43, 41.66)	< 0.0001	2.31 (0.58, 9.27)	0.2358
		Tertile 1	Reference		Reference		Reference	
		Tertile 2	1.77 (1.60, 1.96)	< 0.0001	1.98 (1.78, 2.19)	< 0.0001	1.30 (0.89, 1.90)	0.1734
		Tertile 3	2.29 (2.08, 2.53)	< 0.0001	2.66 (2.40, 2.94)	< 0.0001	1.26 (0.86, 1.84)	0.2433
		*P* for trend	< 0.0001		< 0.0001		0.3665	
**Height**	**CKD**	Height as continuous variable	1.01 (1.01, 1.01)	< 0.0001	1.01 (1.01, 1.01)	< 0.0001	0.99 (0.98, 1.00)	0.0900
		Tertile 1	Reference		Reference		Reference	
		Tertile 2	1.05 (0.98, 1.12)	0.1829	1.07 (1.00, 1.14)	0.0450	0.85 (0.66, 1.09)	0.1935
		Tertile 3	1.28 (1.20, 1.36)	< 0.0001	1.34 (1.26, 1.43)	< 0.0001	0.82 (0.64, 1.05)	0.1106
		*P* for trend	< 0.0001		< 0.0001		0.1229	
	**Albuminuria**	Height as continuous variable	0.98 (0.98, 0.98)	< 0.0001	0.98 (0.98, 0.98)	< 0.0001	0.97 (0.95, 0.98)	< 0.0001
		Tertile 1	Reference		Reference		Reference	
		Tertile 2	0.88 (0.81, 0.94)	0.0005	0.88 (0.82, 0.95)	0.0007	0.70 (0.54, 0.90)	0.0064
		Tertile 3	0.67 (0.62, 0.72)	< 0.0001	0.67 (0.62, 0.73)	< 0.0001	0.45 (0.35, 0.58)	< 0.0001
		*P* for trend	< 0.0001		< 0.0001		< 0.0001	
	**Low-eGFR**	Height as continuous variable	1.05 (1.04, 1.05)	< 0.0001	1.06 (1.05, 1.06)	< 0.0001	1.05 (1.03, 1.06)	< 0.0001
		Tertile 1	Reference		Reference		Reference	
		Tertile 2	1.69 (1.52, 1.89)	< 0.0001	1.84 (1.64, 2.06)	< 0.0001	1.78 (1.23, 2.57)	0.0020
		Tertile 3	3.24 (2.93, 3.58)	< 0.0001	3.89 (3.51, 4.31)	< 0.0001	2.98 (2.10, 4.21)	< 0.0001
		*P* for trend	< 0.0001		< 0.0001		< 0.0001	
**Weight**	**CKD**	Weight as continuous variable	1.01 (1.00, 1.01)	< 0.0001	1.01 (1.00, 1.01)	< 0.0001	1.00 (0.99, 1.01)	0.6614
		Tertile 1	Reference		Reference		Reference	
		Tertile 2	1.03 (0.97, 1.10)	0.3593	1.07 (0.99, 1.14)	0.0526	0.80 (0.62, 1.04)	0.0990
		Tertile 3	1.30 (1.22, 1.39)	< 0.0001	1.39 (1.30, 1.48)	< 0.0001	0.93 (0.71, 1.20)	0.5574
		*P* for trend	< 0.0001		< 0.0001		0.8515	
	**Albuminuria**	Weight as continuous variable	0.99 (0.99, 0.99)	< 0.0001	0.99 (0.99, 0.99)	< 0.0001	0.99 (0.99, 0.99)	0.0031
		Tertile 1	Reference		Reference		Reference	
		Tertile 2	0.70 (0.65, 0.76)	< 0.0001	0.71 (0.65, 0.76)	< 0.0001	0.59 (0.45, 0.78)	0.0002
		Tertile 3	0.80 (0.74, 0.86)	< 0.0001	0.81 (0.75, 0.87)	< 0.0001	0.55 (0.42, 0.72)	< 0.0001
		*P* for trend	< 0.0001		< 0.0001		< 0.0001	
	**Low-eGFR**	Weight as continuous variable	1.02 (1.02, 1.02)	< 0.0001	1.02 (1.02, 1.02)	< 0.0001	1.01 (1.01, 1.02)	< 0.0001
		Tertile 1	Reference		Reference		Reference	
		Tertile 2	2.44 (2.18, 2.72)	< 0.0001	2.76 (2.46, 3.09)	< 0.0001	1.95 (1.32, 2.88)	0.0007
		Tertile 3	3.33 (3.00, 3.70)	< 0.0001	4.10 (3.67, 4.57)	< 0.0001	2.40 (1.64, 3.51)	< 0.0001
		*P* for trend	< 0.0001		< 0.0001		< 0.0001	
**WC**	**CKD**	WC as continuous variable	1.01 (1.01, 1.01)	< 0.0001	1.01 (1.01, 1.02)	< 0.0001	1.01 (1.00, 1.02)	0.0020
		Tertile 1	Reference		Reference		Reference	
		Tertile 2	1.02 (0.96, 1.10)	0.4773	1.07 (0.99, 1.15)	0.0513	0.93 (0.70, 1.23)	0.5970
		Tertile 3	1.62 (1.52, 1.73)	< 0.0001	1.74 (1.63, 1.86)	< 0.0001	1.39 (1.04, 1.85)	0.0250
		*P* for trend	< 0.0001		< 0.0001		0.0034	
	**Albuminuria**	WC as continuous variable	1.01 (1.00, 1.01)	< 0.0001	1.01 (1.00, 1.01)	< 0.0001	1.00 (0.99, 1.01)	0.3116
		Tertile 1	Reference		Reference		Reference	
		Tertile 2	0.77 (0.71, 0.83)	< 0.0001	0.77 (0.71, 0.84)	< 0.0001	0.74 (0.55, 1.01)	0.0560
		Tertile 3	1.22 (1.13, 1.31)	< 0.0001	1.23 (1.14, 1.33)	< 0.0001	1.08 (0.80, 1.47)	0.6066
		*P* for trend	< 0.0001		< 0.0001		0.1734	
	**Low-eGFR**	WC as continuous variable	1.03 (1.02, 1.03)	< 0.0001	1.03 (1.03, 1.03)	< 0.0001	1.02 (1.01, 1.03)	< 0.0001
		Tertile 1	Reference		Reference		Reference	
		Tertile 2	2.15 (1.92, 2.40)	< 0.0001	2.46 (2.19, 2.75)	< 0.0001	2.06 (1.34, 3.16)	0.0009
		Tertile 3	3.37 (3.04, 3.74)	< 0.0001	4.15 (3.72, 4.62)	< 0.0001	2.43 (1.59, 3.73)	< 0.0001
		*P* for trend	< 0.0001		< 0.0001		0.0002	

In sensitivity analysis, WWI, BMI, WHTR, WC, height and weight were converted from continuous variables to categorical variables (tertiles)

^a^OR: Odd ratio

^b^95% CI: 95% confidence interval

^c^Model 1: No covariates were adjusted

^d^Model 2: Adjusted for age, sex, and race

^e^Model 3: Adjusted for sex, age, race, education level, smoking status, serum uric acid, TC, LDL-C, HDL-C, triglycerides, serum total calcium, hypertension, and diabetes status

**Fig. 2:**
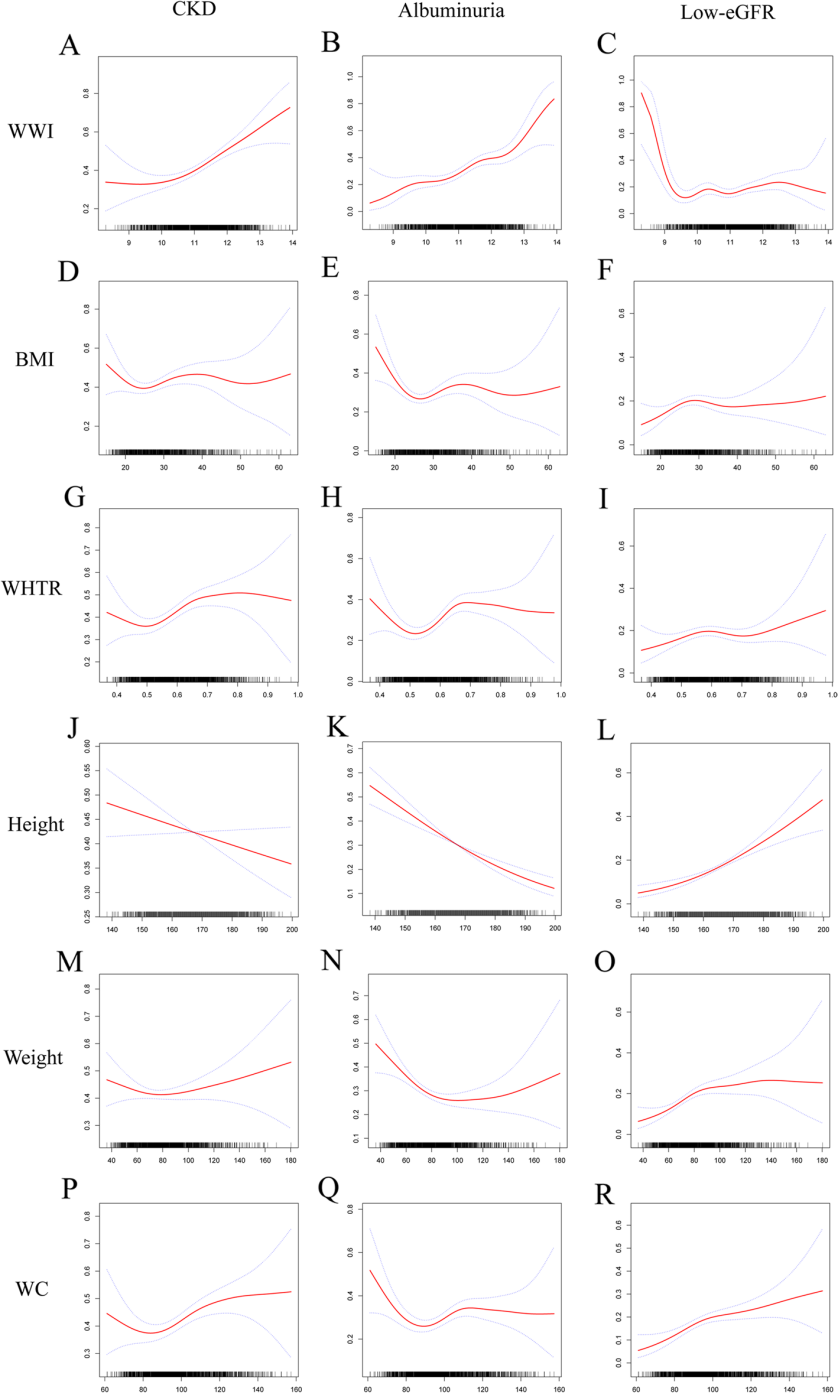
Smooth curve fitting for WWI and other obesity indicators with CKD, albuminuria, and low-eGFR. (**A**) WWI and CKD; (**B**) WWI and albuminuria; (**C**) WWI and low-eGFR; (**D**) BMI and CKD; (**E**) BMI and albuminuria; (**F**) BMI and low-eGFR; (**G**) WHTR and CKD; (**H**) WHTR and albuminuria; (**I**) WHTR and low-eGFR; (**J**) Height and CKD; (**K**) Height and albuminuria; (**L**) Height and low-eGFR; (M) Weight and CKD; (**N**) Weight and albuminuria; (**O**) Weight and low-eGFR; (**P**) WC and CKD; (**Q**) WC and albuminuria; (**R**) WC and low-eGFR

We detected nonlinear relationships of WWI, WHTR, WC, BMI, and weight with CKD by GAM and smooth curve fitting (Fig. 2). In Model 3, the breakpoints were 9.81, 0.49, 79, 20.6 and 67.7, respectively. WWI was positively related to the prevalence of CKD when WWI > 9.81 (OR = 1.52, 95% CI: 1.33, 1.74). To the left of the breakpoint, there was no significant relationship between WWI and CKD (OR = 0.55, 95% CI: 0.25, 1.21) (Table 3). Similarly, there was a nonlinear association between WWI, BMI, WHTR, weight, and WC with CKD (EKFC) (Logarithmic likelihood ratio test *P*-value < 0.05)(Supplementary Figure S1, Supplementary Table S2).

**Table 3:** Threshold effect analysis of WWI and other obesity indicators on CKD, albuminuria, and low-eGFR using a two-piecewise linear regression model in Model 3

	CKD		Albuminuria		Low-eGFR	
	OR^a^ (95%CI^b^)	*P-* value	OR (95%CI)	*P-* value	OR (95%CI)	*P-* value
**WWI**						
Fitting by standard linear model	1.42 (1.26, 1.60)	< 0.0001	1.60 (1.40, 1.82)	< 0.0001	1.10 (0.95, 1.28)	0.2038
Fitting by two-piecewise linear model						
Breakpoint (K)	9.81		12.55		9.58	
OR1(< K)	0.55 (0.25, 1.21)	0.1395	1.52 (1.32, 1.75)	< 0.0001	0.09 (0.03, 0.32)	0.0002
OR2(> K)	1.52 (1.33, 1.74)	< 0.0001	4.29 (1.32, 13.89)	0.0152	1.25 (1.06, 1.47)	0.0078
OR2 / OR1	2.76 (1.19, 6.42)	0.0181	2.82 (0.83, 9.55)	0.0964	13.50 (3.61, 50.48)	0.0001
Logarithmic likelihood ratio test *P*-value	0.020		0.083		< 0.001	
**BMI**						
Fitting by standard linear model	1.01 (0.99, 1.02)	0.2558	1.00 (0.98, 1.02)	0.9579	1.01 (0.99, 1.03)	0.4459
Fitting by two-piecewise linear model						
Breakpoint (K)	20.60		23.9		26.45	
OR1(< K)	0.79 (0.65, 0.96)	0.0167	0.86 (0.80, 0.93)	0.0002	1.11 (1.03, 1.19)	0.0075
OR2(> K)	1.02 (0.99, 1.03)	0.0635	1.02 (1.00, 1.04)	0.0484	0.99 (0.96, 1.01)	0.2834
OR2 / OR1	1.29 (1.05, 1.57)	0.0128	1.18 (1.08, 1.29)	0.0002	0.89 (0.82, 0.97)	0.0097
Logarithmic likelihood ratio test *P*-value	0.013		< 0.001		0.008	
**WHTR**						
Fitting by standard linear model	7.00 (2.41, 20.36)	0.0004	7.19 (2.35, 22.00)	0.0006	2.31 (0.58, 9.27)	0.2358
Fitting by two-piecewise linear model						
Breakpoint (K)	0.49		0.5		0.55	
OR1(< K)	0.01 (0.01, 4.02)	0.1233	0.01 (0.01, 0.23)	0.0144	105.95 (1.04, 175.04)	0.0479
OR2(> K)	13.33 (3.92, 45.29)	< 0.0001	20.21 (5.53, 73.87)	< 0.0001	0.77 (0.12, 5.06)	0.7862
OR2 / OR1	20.17 (1.98, 81.24)	0.0314	50.87 (9.47, 92.6)	0.0017	0.01 (0.01, 2.01)	0.0859
Logarithmic likelihood ratio test *P*-value	0.033		0.002		0.081	
**Height**						
Fitting by standard linear model	0.99 (0.98, 1.00)	0.0900	0.97 (0.95, 0.98)	< 0.0001	1.05 (1.03, 1.06)	< 0.0001
Fitting by two-piecewise linear model						
Breakpoint (K)	151		165.7		175.5	
OR1(< K)	0.95 (0.86, 1.05)	0.3241	0.97 (0.95, 1.00)	0.0185	1.06 (1.04, 1.08)	< 0.0001
OR2(> K)	0.99 (0.98, 1.00)	0.2019	0.96 (0.94, 0.98)	< 0.0001	1.02 (0.98, 1.06)	0.4167
OR2 / OR1	1.04 (0.94, 1.15)	0.4124	0.98 (0.95, 1.02)	0.3749	0.96 (0.91, 1.01)	0.1097
Logarithmic likelihood ratio test *P*-value	0.414		0.374		0.107	
**Weight**						
Fitting by standard linear model	1.00 (0.99, 1.01)	0.6614	0.99 (0.99, 0.99)	0.0031	1.01 (1.01, 1.02)	< 0.0001
Fitting by two-piecewise linear model						
Breakpoint (K)	67.7		82.2		81.8	
OR1(< K)	0.98 (0.96, 0.99)	0.0241	0.97 (0.96, 0.99)	< 0.0001	1.04 (1.02, 1.06)	< 0.0001
OR2(> K)	1.01 (1.00, 1.01)	0.0766	1.00 (0.99, 1.01)	0.3273	1.00 (0.99, 1.01)	0.7520
OR2 / OR1	1.03 (1.01, 1.05)	0.0150	1.03 (1.01, 1.05)	0.0004	0.96 (0.94, 0.99)	0.0015
Logarithmic likelihood ratio test *P*-value	0.015		< 0.001		0.001	
**WC**						
Fitting by standard linear model	1.01 (1.00, 1.02)	0.0020	1.00 (0.99, 1.01)	0.3116	1.02 (1.01, 1.03)	< 0.0001
Fitting by two-piecewise linear model						
Breakpoint (K)	79		80		90.5	
OR1(< K)	0.95 (0.90, 0.99)	0.0394	0.92 (0.88, 0.97)	0.0012	1.07 (1.03, 1.10)	0.0006
OR2(> K)	1.01 (1.01, 1.02)	0.0001	1.01 (1.00, 1.02)	0.0170	1.01 (0.99, 1.02)	0.1405
OR2 / OR1	1.07 (1.01, 1.14)	0.0143	1.09 (1.04, 1.15)	0.0007	0.95 (0.91, 0.99)	0.0084
Logarithmic likelihood ratio test *P*-value	0.015		< 0.001		0.006	

Adjusted for sex, age, race, education level, smoking status, serum uric acid, TC, LDL-C, HDL-C, triglycerides, serum total calcium, hypertension, and diabetes status

^a^*OR* Odd ratio

^b^*95% CI* 95% confidence interval

### Association between WWI and albuminuria

We found positive associations between WWI and WHTR with albuminuria (Table 2). In Model 3, the prevalences of albuminuria increased by 60% and 6.19-fold for each one-unit increase in WWI and WHTR (WWI: OR = 1.60; 95% CI: 1.40, 1.82; WHTR: OR = 7.19; 95% CI: 2.35, 22.00). In contrast, higher levels of height versus weight were related to a lower prevalence of albuminuria in Model 3 (Height: OR = 0.97; 95% CI: 0.95, 0.98; Weight: OR = 0.99; 95% CI: 0.99, 0.99).

We detected nonlinear relationships of BMI, WHTR, weight and WC with albuminuria by smooth curve fitting (Fig. 2). Their breakpoints were 23.9, 0.5, 82.2 and 80, respectively (Logarithmic likelihood ratio test *P*-value < 0.05) (Table 3). We did not find a nonlinear relationship between WWI and albuminuria.

### Association between WWI and low-eGFR

The relationships between WWI and other obesity indicators with low-eGFR were also evaluated using three distinct models (Table 2). We did not find a significant association of WWI with low-eGFR in Model 3 (OR = 1.10, 95% CI: 0.95, 1.28) (Table 2). But we found positive associations of height, weight and WC with low-eGFR. We found similar associations between WWI and other obesity indicators with low-eGFR (EKFC) (Supplementary Table S1).

We did not find a significant association of WWI with low-eGFR by the weighted multivariable regression model in Model 3 (Table 2). However, we detected an L-shaped relationship of WWI with low-eGFR by smooth curve fitting (Fig. 2). The breakpoint was 9.58. WWI was negatively related to low-eGFR when WWI < 9.58 (OR = 0.09, 95% CI: 0.03, 0.32). On the right side of the breakpoint, the prevalence of low-eGFR increased by 25% for each unit increase in WWI (OR = 1.25, 95% CI: 1.06, 1.47).

### Subgroup analysis

**Fig. 3:**
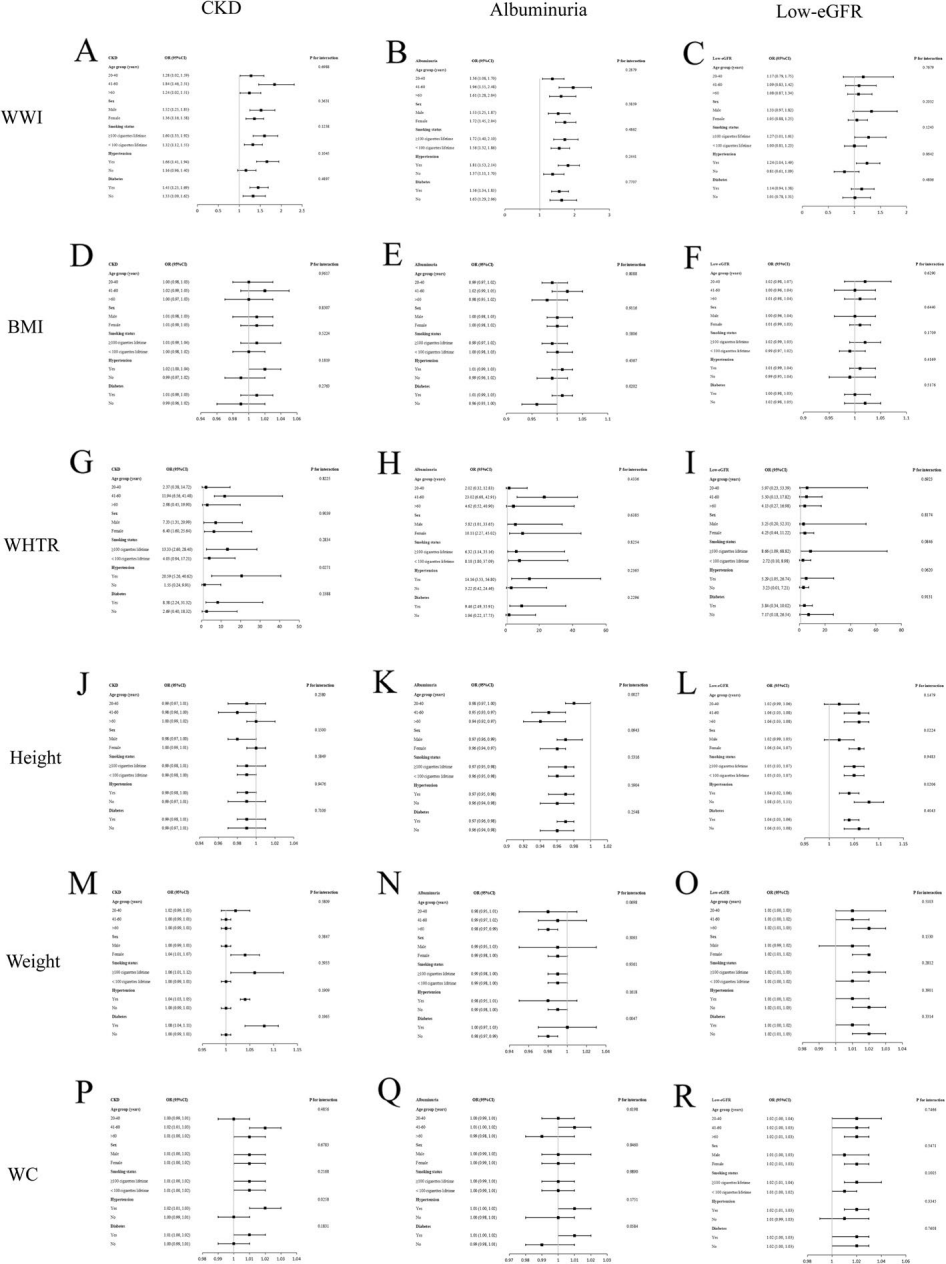
Subgroup analysis for the associations of WWI and other obesity indicators with CKD, albuminuria, and low-eGFR. (**A**) WWI and CKD; (**B**) WWI and albuminuria; (**C**) WWI and low-eGFR; (**D**) BMI and CKD; (**E**) BMI and albuminuria; (**F**) BMI and low-eGFR; (**G**) WHTR and CKD; (**H**) WHTR and albuminuria; (**I**) WHTR and low-eGFR; (**J**) Height and CKD; (**K**) Height and albuminuria; (**L**) Height and low-eGFR; (**M**) Weight and CKD; (**N**) Weight and albuminuria; (**O**) Weight and low-eGFR; (**P**) WC and CKD; (**Q**) WC and albuminuria; (**R**) WC and low-eGFR

Our results indicated that the correlations between WWI and other obesity indicators with CKD are not consistent (Fig. 3). According to the interaction tests, age, sex, smoking status, hypertension, and diabetes did not substantially influence the relationship between WWI and CKD across strata (all *p* for interaction > 0.05). The relationships between WHTR and WC with CKD depended on the hypertensive status and may be applicable to hypertensive populations (Fig. 3). Interaction tests showed that the relationships between WWI and other obesity indicators with CKD (EKFC) were applicable in different populations (all *p* for interaction > 0.05) (Supplementary Figure S2).

A positive association between WWI and albuminuria was found in all subgroups (all *p* < 0.05) (Fig. 3). The relationships between WWI, WHTR, and WC with albuminuria were not substantially associated in interaction tests with various stratifications, demonstrating that these associations were the same across population contexts (all *p* for interaction > 0.05) (Fig. 3).

Interaction tests showed that the relationships between WWI, BMI, WHTR, weight, and WC with low-eGFR were not affected by the above stratification factors (all *p* for interaction > 0.05) (Fig. 3).

### ROC analysis

**Fig. 4:**
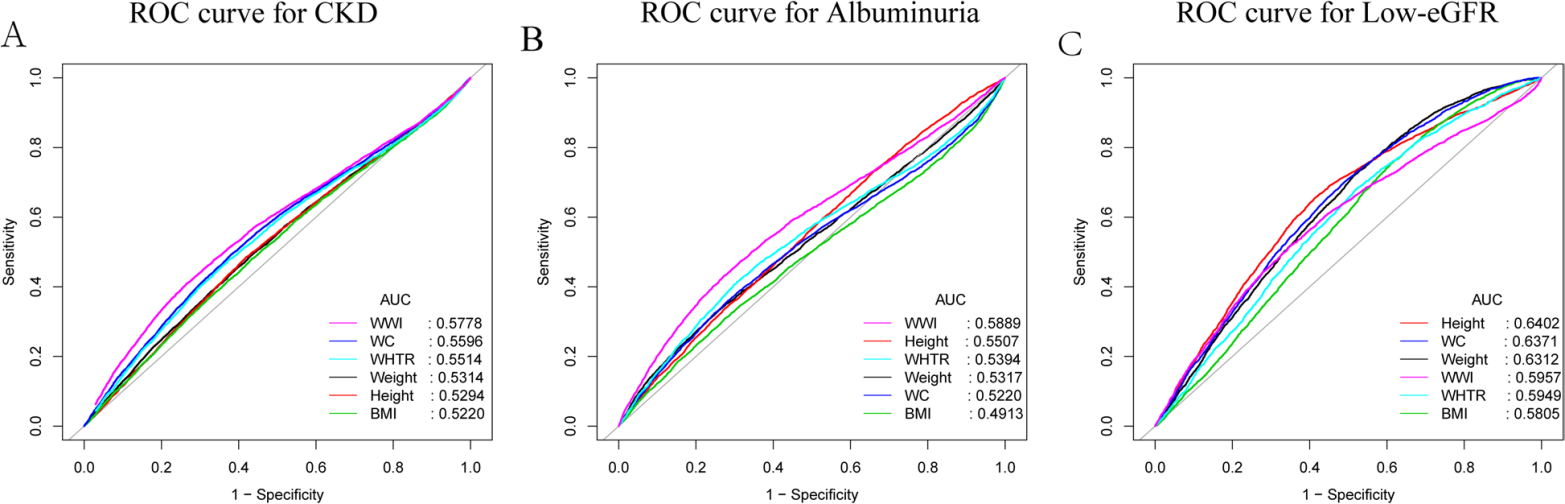
ROC curves and the AUC values of the six obesity indicators (WWI, BMI, WHTR, WC, height, and weight) in diagnosing CKD, albuminuria and low-eGFR. (**A**) Six obesity indicators were assessed to identify CKD. (**B**) Six obesity indicators were assessed to identify albuminuria. (**C**) Six obesity indicators were assessed to identify low-eGFR

We calculated the AUC values to compare the predictive accuracy of WWI with other obesity indicators (BMI, WHTR, WC, height, and weight) for CKD, albuminuria, and low-eGFR (Fig. 4). We found that the AUC values of WWI were higher than the other 5 obesity indicators in predicting CKD and albuminuria (CKD: AUC (95% CI): 0.5778 (0.5699–0.5857); albuminuria: AUC (95% CI): 0.5889 (0.5794–0.5983)). Moreover, the difference in AUC values between WWI and other obesity indicators was statistically significant (all *p* < 0.05), suggesting that WWI may be a better predictive indicator for CKD and albuminuria than BMI, WHTR, weight, height, and WC (Table 4). Similarly, WWI was significantly better than other obesity indicators in predicting CKD (EKFC) (AUC (95% CI): 0.5653 (0.5578–0.5728)(all *p* < 0.05) (Supplementary Figure S3, Supplementary Table 3). Additionally, Height was the best predictor in predicting low-eGFR (AUC (95% CI): 0.6402 (0.6302–0.6502)) (Table 4).

**Table 4:** Comparison of AUC values between WWI and other obesity indicators

Test	AUC^1^	95%CI^2^ low	95%CI upp	Best threshold	Specificity	Sensitivity	*P* for different in AUC
**CKD**							
WWI	0.5778	0.5699	0.5857	11.3439	0.7342	0.4099	Reference
BMI	0.5220	0.5143	0.5297	27.405	0.5677	0.4783	< 0.0001
WHTR	0.5514	0.5436	0.5593	0.5880	0.6253	0.4785	< 0.0001
Height	0.5294	0.5218	0.5371	167.75	0.5804	0.4855	< 0.0001
Weight	0.5314	0.5237	0.5391	78.55	0.6080	0.4503	< 0.0001
WC	0.5596	0.5518	0.5674	97.45	0.6192	0.4931	< 0.0001
**Albuminuria**							
WWI	0.5889	0.5794	0.5983	11.3446	0.7278	0.4312	Reference
BMI	0.4913	0.4816	0.5010	30.445	0.7252	0.3088	< 0.0001
WHTR	0.5394	0.5296	0.5492	0.6117	0.7013	0.4061	< 0.0001
Height	0.5507	0.5418	0.5596	170.55	0.3424	0.7289	< 0.0001
Weight	0.5317	0.5221	0.5412	61.25	0.7645	0.3088	< 0.0001
WC	0.5220	0.5122	0.5318	102.65	0.7166	0.3604	< 0.0001
**Low-eGFR**							
WWI	0.5957	0.5847	0.6066	11.1439	0.6494	0.5183	< 0.0001
BMI	0.5805	0.5711	0.5900	24.655	0.3895	0.7546	< 0.0001
WHTR	0.5949	0.5850	0.6047	0.5491	0.4768	0.6834	< 0.0001
Height	0.6402	0.6302	0.6502	167.75	0.5878	0.6538	Reference
Weight	0.6312	0.6220	0.6404	72.27	0.4874	0.7177	< 0.0001
WC	0.6371	0.6277	0.6465	92.65	0.5097	0.7015	< 0.0001

^1^AUC: area under the curve

^2^95% CI: 95% confidence interval

### Association between WWI and eGFR

We also analyzed the association of WWI with eGFR. WWI was positively correlated with eGFR in Model 4 (Supplementary Table S4). We detected a nonlinear relationship and a saturation effect of WWI with eGFR by GAM and smooth curve fitting (Supplementary Figure S4). The breakpoint was 10.62 (Supplementary Table S5). Subgroup analysis revealed that the relationship of WWI with eGFR was dependent on age, smoking status, and hypertension (Supplementary Figure S5). We also found similar results for the relationship of WWI with eGFR (EKFC) (Supplementary Tables S6 and S7, Supplementary Figures S6 and S7).

## Discussion

In this cross-sectional study including 40,421 adults, we found a positive association between WWI and CKD. Through smooth curve fitting, we identified a threshold effect of the nonlinear relationship between WWI and CKD, which was determined to be a breakpoint of 9.81 cm/√kg. Additionally, we discovered a positive correlation between WWI and albuminuria. There was an L-shaped association between WWI and low-eGFR. Subgroup analysis and interaction tests indicated no significant differences in the associations between WWI with CKD, albuminuria, and low-eGFR among different populations. ROC analysis showed that WWI was the best predictor of CKD and albuminuria when compared to other obesity indicators (BMI, WHTR, WC, height, and weight). Additionally, higher height was associated with a higher prevalence of low-eGFR. ROC analysis showed that height was the best predictor of low-eGFR. In conclusion, we need to focus on the importance of high WWI and height levels in assessing kidney health in US adults.

Previous research has mostly focused on the links between other obesity indicators and CKD. Previous studies have made mixed statements about the association between BMI and CKD [17–20]. In contrast, we found a nonlinear relationship of BMI with CKD. Below 20.60, BMI was negatively associated with CKD. Above the breakpoint, there was no significant association between BMI and CKD. This suggests that high BMI may be protective for CKD. This may be due to the limitation of BMI in distinguishing muscle mass from fat mass. Those of the same height but with a greater degree of body muscularity may have the same BMI as those with a high fat mass [21]. And high fat and low muscle mass are strongly associated with the risk of developing CKD [22, 23]. This is further validated by our study, in which ROC analysis showed that BMI was the poorest predictor of CKD, albuminuria, and low-eGFR compared with other obesity indicators. WC reflects abdominal fat accumulation but does not account for the effect of height on body fat distribution. The use of WC alone may overestimate the risk of obesity in taller or underestimate the risk of obesity in people with short stature [21]. Previous studies have shown that the association between WC and CKD is not strong [24, 25]. Our study agrees with this view, with the prevalences of CKD and low-eGFR increasing by only 1% and 2% for each unit increase in WC. In conclusion, neither WC nor BMI can be used as the best predictor of renal function in the US adult population. Previous studies have shown that WHtR is strongly associated with renal function [24, 26, 27]. Our study also showed that the prevalences of CKD and albuminuria increased 6-fold and 6.19-fold, respectively, for each unit increase in WHtR. Also, there were nonlinear associations between WHtR with CKD and albuminuria. However, unlike previous studies, we did not find a significant association between WHtR and low-eGFR [21, 26]. We think that various demographic factors, such as region, race, population, sample size, and eGFR calculation method, can produce various outcomes. Our study also found that height was positively associated with low-eGFR. By ROC analysis, height was the best predictor of low-eGFR. This may be due to the fact that height is generally higher in men than in women. And previous studies have shown that men were more likely to have worsened renal function due to testosterone and sex hormones [28, 29]. Therefore, American adults with higher height should be aware of kidney health.

This is the first investigation that we are aware of that examines the connection between WWI and CKD. There are few prior investigations on the connection between WWI and kidney function. Only one study with 36,921 US adults found that those with higher WWI had a higher likelihood of having albuminuria [11]. This was also confirmed in our study. In WWI, the prevalence of albuminuria increased by 60% for every unit increment. In a study including 24,791 Chinese participants, Kang et al. discovered that a higher visceral fat area (VFA) was linked to a higher risk of CKD [30]. In a cross-sectional study involving 35,018 US adults, Qin et al. found that participants with higher visceral obesity index (VAI) had an increased risk of developing CKD and albuminuria [31]. According to our findings, which are in line with earlier research, WWI was found to be positively linked with CKD. The non-linear association between WWI and CKD was also found to have a threshold effect in the current investigation, with a breakpoint of 9.81 cm/kg. WWI is negatively but not statistically significantly linked with CKD when it is less than 9.81. WWI and CKD were positively associated on the right side of the breakpoint. In other words, the prevalence of CKD considerably rose when WWI > 9.81. In conclusion, WWI may have a significant negative impact on kidney function. Previous studies have also observed the superiority of WWI. Compared to BMI, WC, WHtR, and a body shape index(ABSI), WWI is the best predictor of cardiovascular disease [6]. Qin et al. showed that WWI has a higher correlation with albuminuria than BMI and WC [11]. This is attributed to WWI as a new obesity indicator that can effectively distinguish between fat mass and muscle mass [7, 8]. Our study agrees with this view, and ROC analysis showed that WWI was the best obesity indicator for predicting CKD and albuminuria compared with other obesity indicators (BMI, WHTR, WC, height, and weight). Thus, WWI can be deemed a more precise and all-encompassing measure of obesity, with the added advantage of being low-cost and easily accessible. In conclusion, it holds great promise for predicting kidney health in American adults.

Interestingly, the weighted multivariable regression model showed that WWI was not significantly associated with low-eGFR in Model 3. However, we found an L-shaped association between the two in the smooth curve fitting. When WWI < 9.58, WWI was negatively correlated with low-eGFR. On the right side of the breakpoint, the prevalence of low-eGFR increased by 25% for each unit increase in WWI. That is, the prevalence of low-eGFR was lowest at WWI = 9.58. We further evaluated the association between WWI and eGFR. After adjusting for albuminuria, WWI was positively associated with eGFR. And there was a nonlinear association and saturation effect between the two. Higher WWI was associated with higher eGFR at WWI < 10.62. Whereas, on the right side of the breakpoint, the two were not significantly associated. In conclusion, WWI was a favorable factor for eGFR. This was further validated by our ROC analysis, which showed that WWI was not the best predictor of low-eGFR compared to other obesity indicators.

Compared to people without diabetes, research has shown that participants with diabetes have a significantly higher prevalence of CKD [3]. Our study provides supportive evidence for this view, as subgroup analysis showed that for each unit increase in WWI, participants with diabetes exhibited a higher prevalence of CKD than those without diabetes. Our subgroup analysis also revealed that male participants were more likely to develop CKD. This finding has also been confirmed by previous studies [32]. This may be related to unhealthy lifestyle habits in males, as well as the protective effect of estrogen or the destructive effect of testosterone [33]. Additionally, we found that the effects of age, sex, smoking status, hypertension, or diabetes on the associations between WWI with CKD, albuminuria, and low-eGFR were not statistically significant. These associations might be applicable to various populations. These findings support and provide additional evidence for the harm that WWI caused to kidneys.

The relationship between WWI and CKD may be influenced by inflammation and insulin resistance. Adiposity accumulation can increase the expression of pro-inflammatory adipokines like adiponectin while decreasing the expression of anti-inflammatory adipokines like interleukin-6 (IL-6), tumor necrosis factor-α (TNF-α), and transforming growth factor-β (TGF-β) [34]. Additionally, this buildup has the potential to activate the renin–angiotensin–aldosterone system (RAAS), which can result in hypertension and insulin resistance, both of which are known to be risk factors for kidney injury [18, 35]. Additionally, glomerular hyperperfusion, hypertension, and even functional loss might emerge from central fat distribution relative to effective kidney plasma flow, which can raise the glomerular filtration rate and result in an elevated filtration fraction [36].

Our research possesses various advantages. Firstly, our research relies on NHANES data, a national population-based survey that follows a strict study protocol and quality control measures. Secondly, our large sample size and adjustment for confounding covariates enhance the reliability and representativeness of our study. Given its computational simplicity, WWI can be a practical tool for managing and intervening in CKD in clinical practice. Our study does, however, have certain flaws. First instance, establishing a causal connection between WWI and CKD was impossible due to the cross-sectional design. Second, while we adjusted for numerous important covariates, we cannot eliminate the impact of other possible confounding variables. Third, because NHANES is a cross-sectional survey of the US population, it may be difficult to extrapolate our results to the general population or other ethnic groups.

## Conclusion

WWI is the best obesity indicator to predict CKD and albuminuria compared to other obesity indicators (BMI, WHTR, WC, height, and weight). WWI and CKD and albuminuria were found to be positively correlated. Furthermore, height had the strongest ability to predict low-eGFR. Therefore, the importance of WWI and height in assessing kidney health in US adults should be emphasized. More comprehensive prospective studies are necessary to support the authors' findings.

### Supplementary Information

**Additional file 1.** **Supplementary Figure S1.** Smooth curve fitting for WWI and other obesity indicators with CKD(EKFC) and low-eGFR(EKFC). (A) WWI and CKD(EKFC); (B) WWI and low-eGFR(EKFC); (C) BMI and CKD(EKFC); (D) BMI and low-eGFR(EKFC); (E) WHTR and CKD(EKFC); (F) WHTR and low-eGFR(EKFC); (G) Height and CKD(EKFC); (H) Height and low-eGFR(EKFC); (I) Weight and CKD(EKFC); (J) Weight and low-eGFR(EKFC); (K) WC and CKD(EKFC); (L) WC and low-eGFR(EKFC).

**Additional file 2.** **Supplementary Figure S2.** Subgroup analysis for the associations of WWI and other obesity indicators with CKD(EKFC) and low-eGFR(EKFC). (A) WWI and CKD(EKFC); (B) WWI and low-eGFR(EKFC); (C) BMI and CKD(EKFC); (D) BMI and low-eGFR(EKFC); (E) WHTR and CKD(EKFC); (F) WHTR and low-eGFR(EKFC); (G) Height and CKD(EKFC); (H) Height and low-eGFR(EKFC); (I) Weight and CKD(EKFC); (J) Weight and low-eGFR(EKFC); (K) WC and CKD(EKFC); (L) WC and low-eGFR(EKFC).

**Additional file 3.** **Supplementary Figure S3.** ROC curves and the AUC values of the six obesity indicators(WWI, BMI, WHTR, WC, height, and weight) in diagnosing CKD(EKFC) and low-eGFR(EKFC). (A) Six obesity indicators were assessed to identify CKD(EKFC). (B) Six obesity indicators were assessed to identify low-eGFR(EKFC).

**Additional file 4.** **Supplementary Figure S4.** Smooth curve fitting for WWI and eGFR.

**Additional file 5.** **Supplementary Figure S5.** Subgroup analysis for the association of WWI and eGFR.

**Additional file 6.** **Supplementary Figure S6.** Smooth curve fitting for WWI and eGFR(EKFC).

**Additional file 7.** **Supplementary Figure S7.** Subgroup analysis for the association of WWI and eGFR(EKFC).

**Additional file 8.** **Supplementary Table S1.** Associations between WWI and other obesity indicators with CKD (EKFC) and low-eGFR (EKFC).

**Additional file 9.** **Supplementary Table S2. **Threshold effect analysis of WWI and other obesity indicators on CKD(EKFC) and low-eGFR(EKFC) using a two-piecewise linear regression model in Model 3.

**Additional file 10.** **Supplementary Table S3.** Comparison of AUC values between WWI and other obesity indicators.

**Additional file 11.** **Supplementary Table S4.** Association between WWI and eGFR.

**Additional file 12.** **Supplementary Table S5.** Threshold effect analysis of WWI on eGFR using a two-piecewise linear regression model in Model 1 and Model 4.

**Additional file 13.** **Supplementary Table S6.** Association between WWI and eGFR (EKFC).

**Additional file 14.** **Supplementary Table S7.** Threshold effect analysis of WWI on eGFR (EKFC) using a two-piecewise linear regression model in Model 1 and Model 4.

## Data Availability

Publicly available datasets were analyzed in this study. This data can be found here: https://www.cdc.gov/nchs/nhanes/.

## Abbreviations

WWI: Weight-adjusted-waist index
CKD: Chronic kidney disease
NHANES: National Health and Nutrition Examination Survey
eGFR: Estimated glomerular filtration rate
ACR: Albumin-to-creatinine ratio
BMI: Body mass index
WHtR: Waist-to-height ratio
WC: Waist circumference
ROC: Receiver operating characteristic
AUC: Area under the curve
DALYs: Disability-adjusted life years
NCHS: National Center for Health Statistics
MEC: Mobile examination center
CKD-EPI: Chronic Kidney Disease Epidemiology Collaboration
EKFC: European Kidney Function Consortium
GED: General educational development
TC: Total cholesterol
HDL-C: High-density lipoprotein cholesterol
LDL-C: Low-density lipoprotein cholesterol
HbA1c: Hemoglobin A1c
CDC: Centers for Disease Control and Prevention
GAM: Generalized additive models
VFA: Visceral fat area
VAI: Visceral obesity index
ABSI: A body shape index
IL-6: Interleukin-6
TNF-α: Tumor necrosis factor-α
TGF-β: Transforming growth factor-β
RAAS: Renin–angiotensin–aldosterone system
