# Supplementary figures and images for: Association between weight-adjusted-waist index and chronic kidney disease: a cross-sectional study
Source: BMC Nephrol. 2023 Sep 11;24:266. doi: 10.1186/s12882-023-03316-w (PMC10494374; doi:10.1186/s12882-023-03316-w)

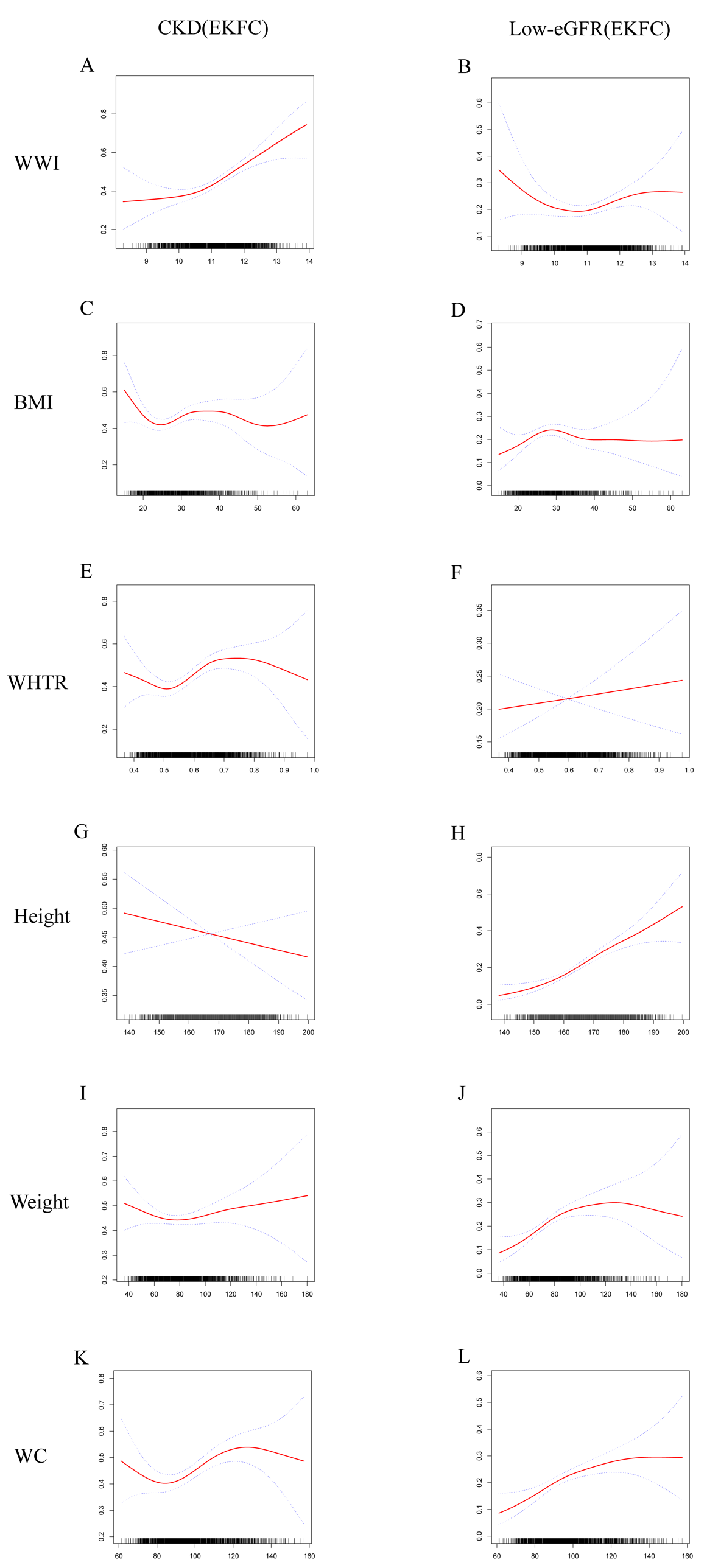

Supplement: Supplementary file 1 — Additional file 1. Supplementary Figure S1. Smooth curve fitting for WWI and other obesity indicators with CKD(EKFC) and low-eGFR(EKFC). (A) WWI and CKD(EKFC); (B) WWI and low-eGFR(EKFC); (C) BMI and CKD(EKFC); (D) BMI and low-eGFR(EKFC); (E) WHTR and CKD(EKFC); (F) WHTR and low-eGFR(EKFC); (G) Height and CKD(EKFC); (H) Height and low-eGFR(EKFC); (I) Weight and CKD(EKFC); (J) Weight and low-eGFR(EKFC); (K) WC and CKD(EKFC); (L) WC and low-eGFR(EKFC). [file 12882_2023_3316_MOESM1_ESM.tif]

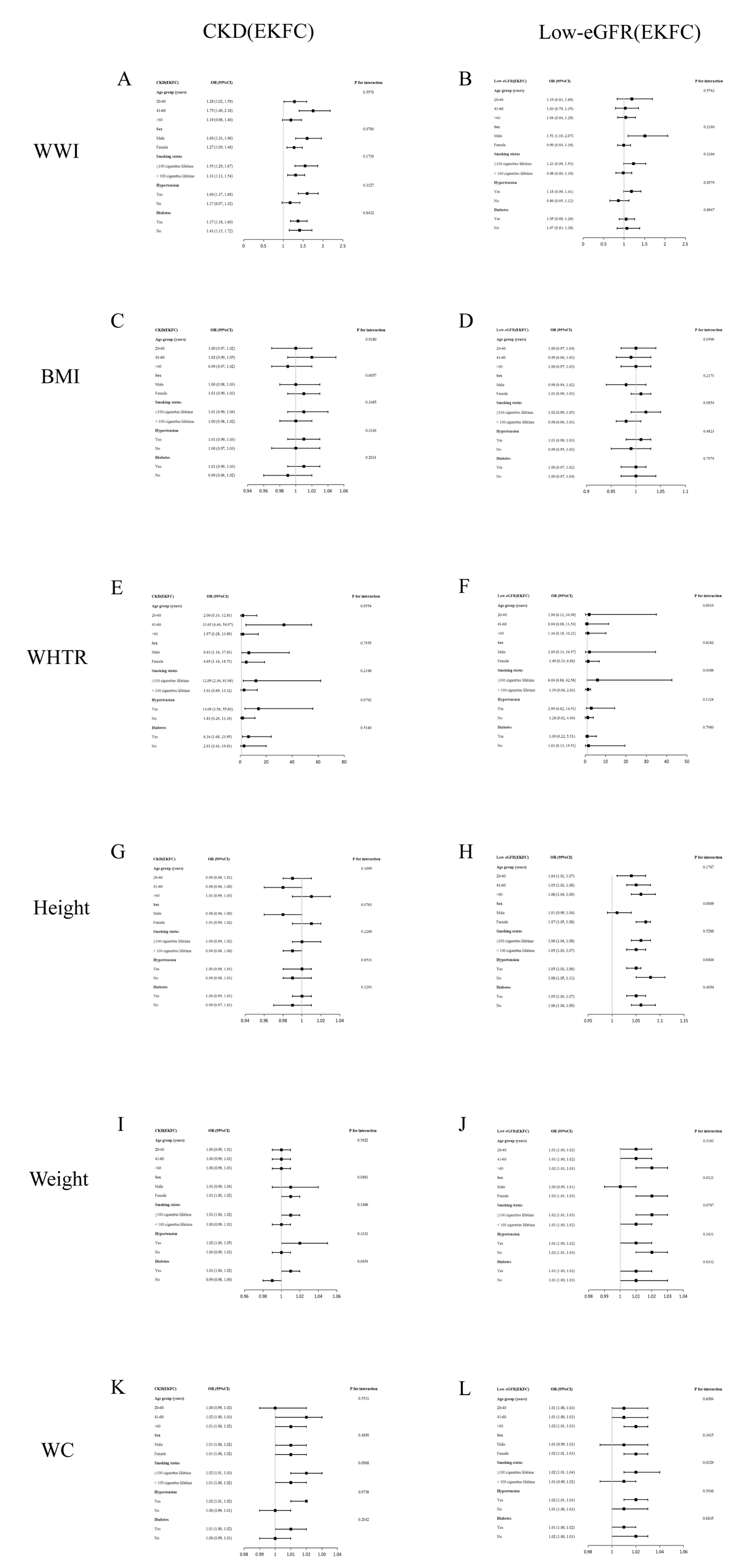

Supplement: Supplementary file 2 — Additional file 2. Supplementary Figure S2. Subgroup analysis for the associations of WWI and other obesity indicators with CKD(EKFC) and low-eGFR(EKFC). (A) WWI and CKD(EKFC); (B) WWI and low-eGFR(EKFC); (C) BMI and CKD(EKFC); (D) BMI and low-eGFR(EKFC); (E) WHTR and CKD(EKFC); (F) WHTR and low-eGFR(EKFC); (G) Height and CKD(EKFC); (H) Height and low-eGFR(EKFC); (I) Weight and CKD(EKFC); (J) Weight and low-eGFR(EKFC); (K) WC and CKD(EKFC); (L) WC and low-eGFR(EKFC). [file 12882_2023_3316_MOESM2_ESM.tif]

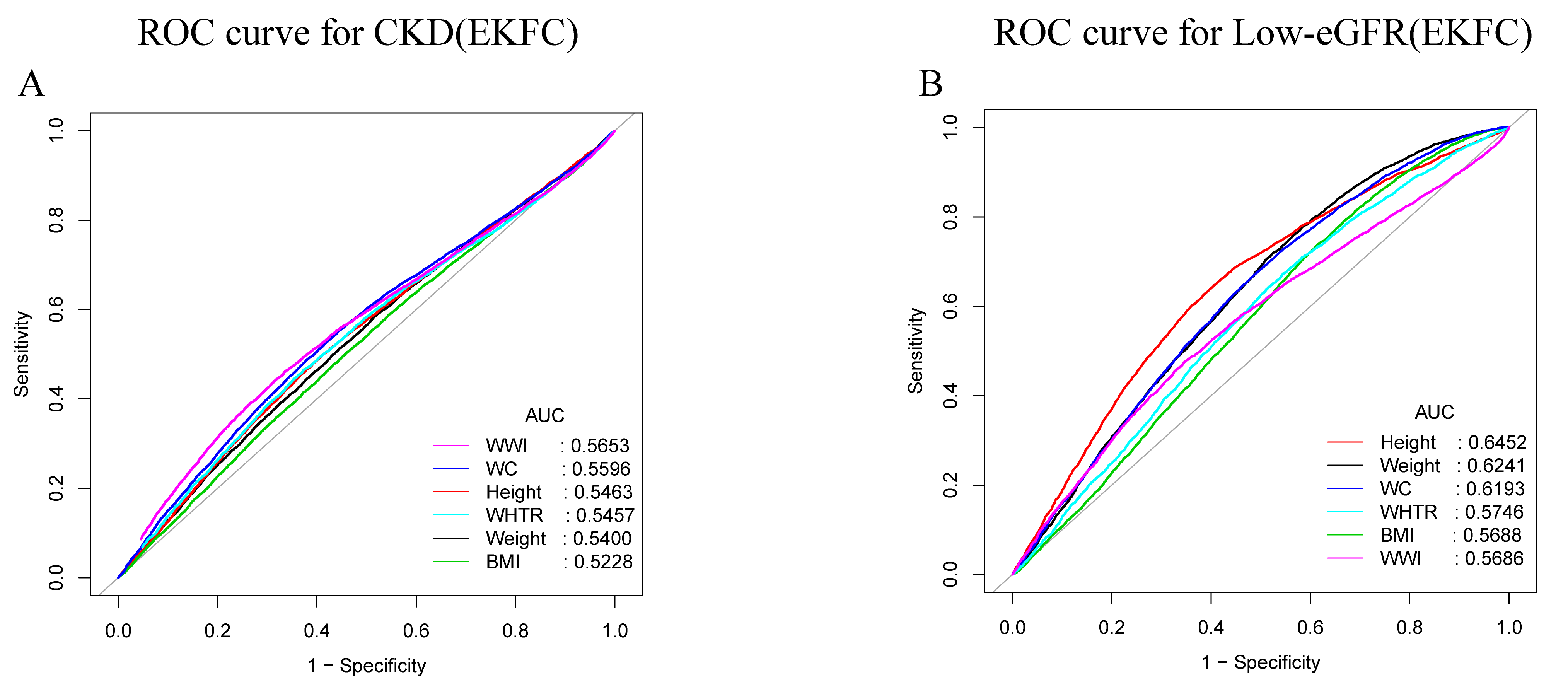

Supplement: Supplementary file 3 — Additional file 3. Supplementary Figure S3. ROC curves and the AUC values of the six obesity indicators(WWI, BMI, WHTR, WC, height, and weight) in diagnosing CKD(EKFC) and low-eGFR(EKFC). (A) Six obesity indicators were assessed to identify CKD(EKFC). (B) Six obesity indicators were assessed to identify low-eGFR(EKFC). [file 12882_2023_3316_MOESM3_ESM.tif]

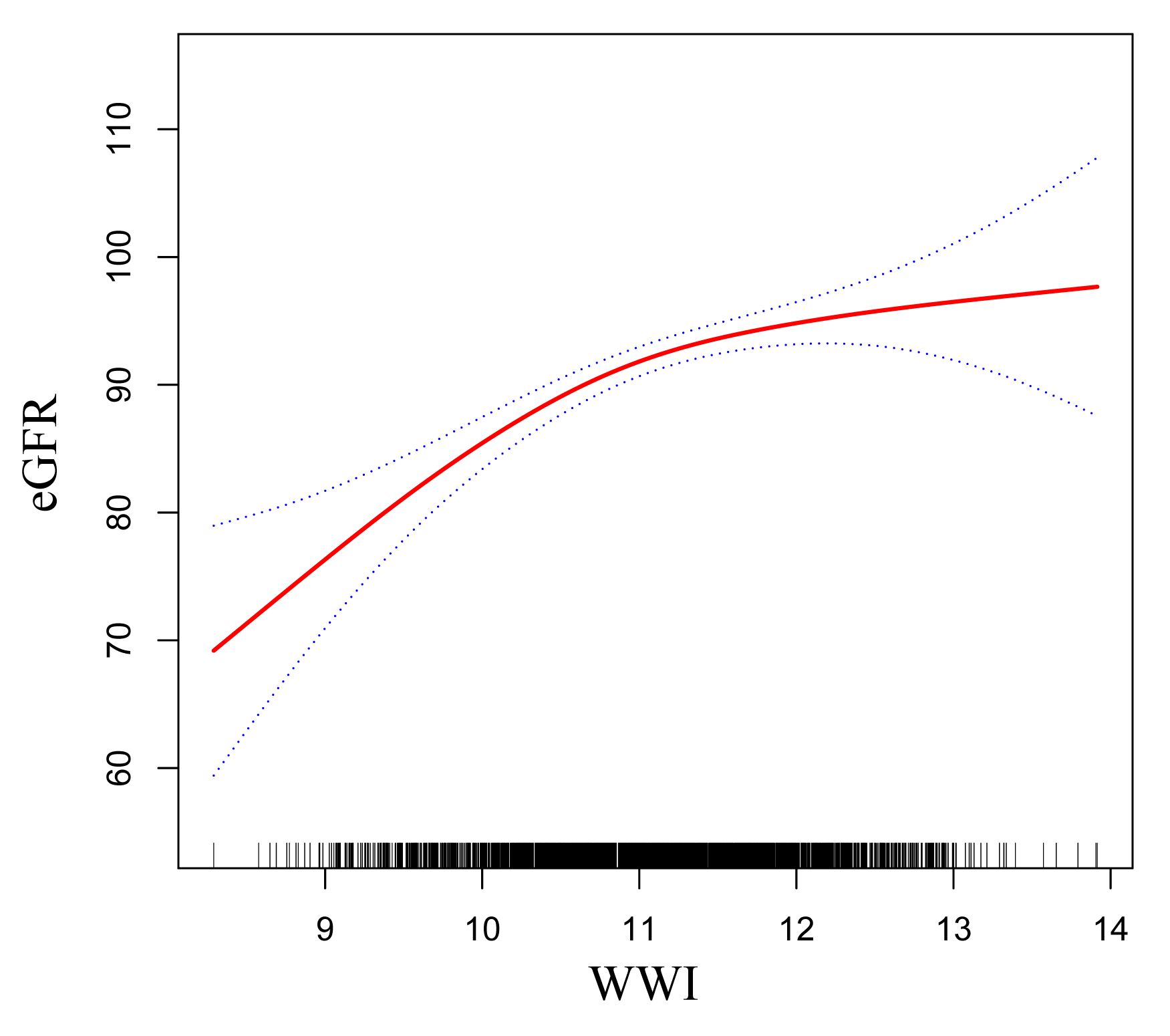

Supplement: Supplementary file 4 — Additional file 4. Supplementary Figure S4. Smooth curve fitting for WWI and eGFR. [file 12882_2023_3316_MOESM4_ESM.tif]

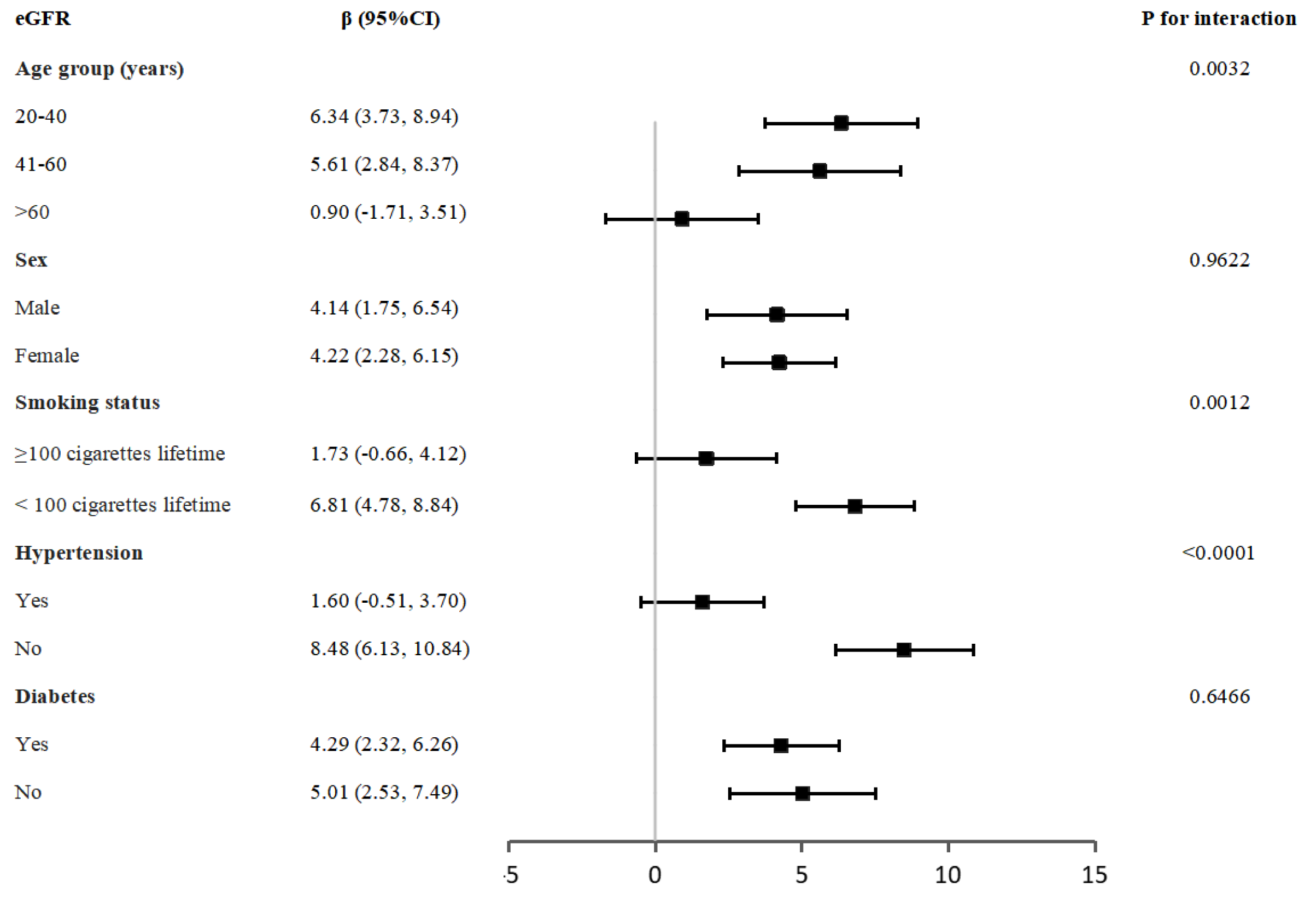

Supplement: Supplementary file 5 — Additional file 5. Supplementary Figure S5. Subgroup analysis for the association of WWI and eGFR. [file 12882_2023_3316_MOESM5_ESM.tif]

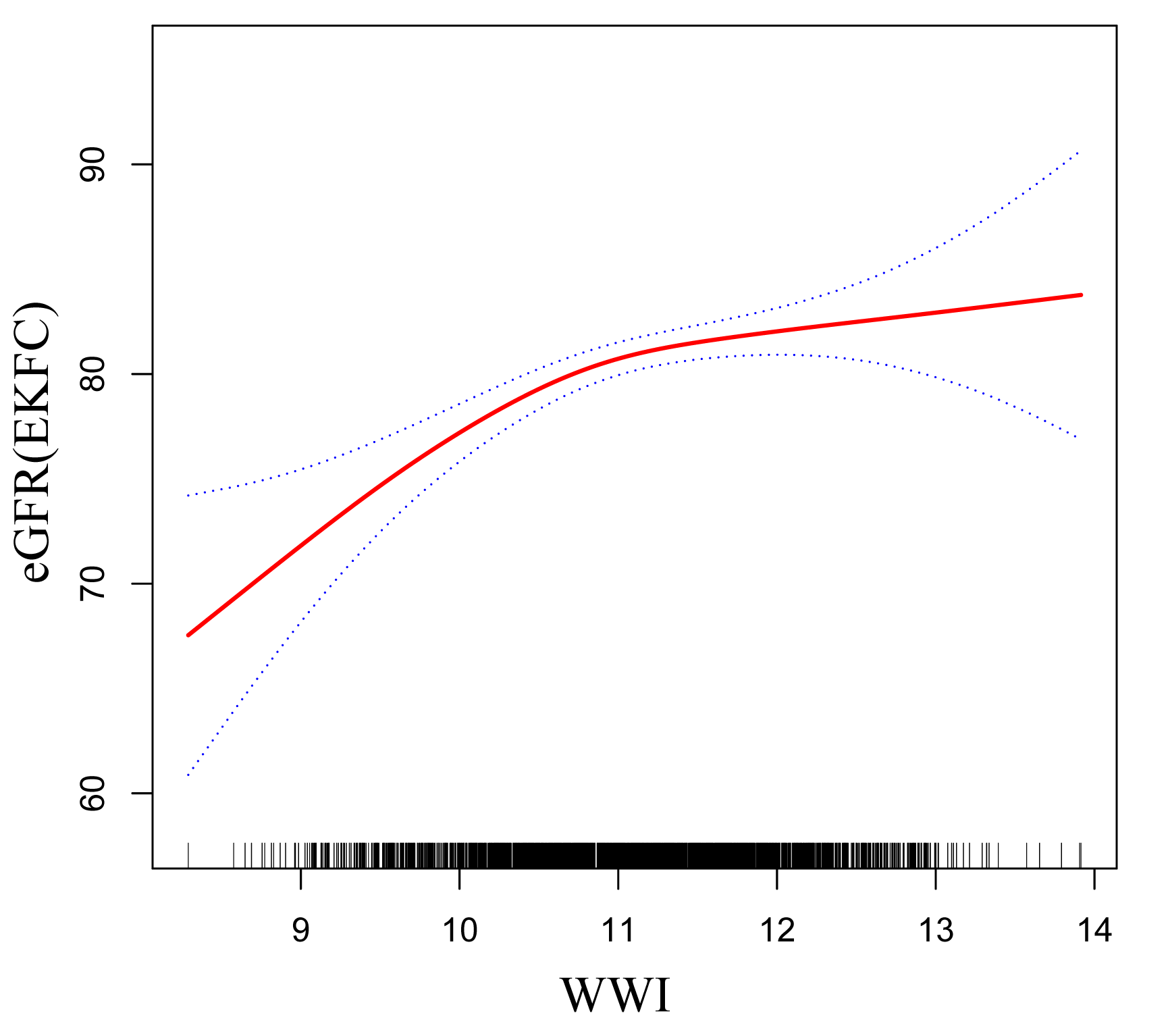

Supplement: Supplementary file 6 — Additional file 6. Supplementary Figure S6. Smooth curve fitting for WWI and eGFR(EKFC). [file 12882_2023_3316_MOESM6_ESM.tif]

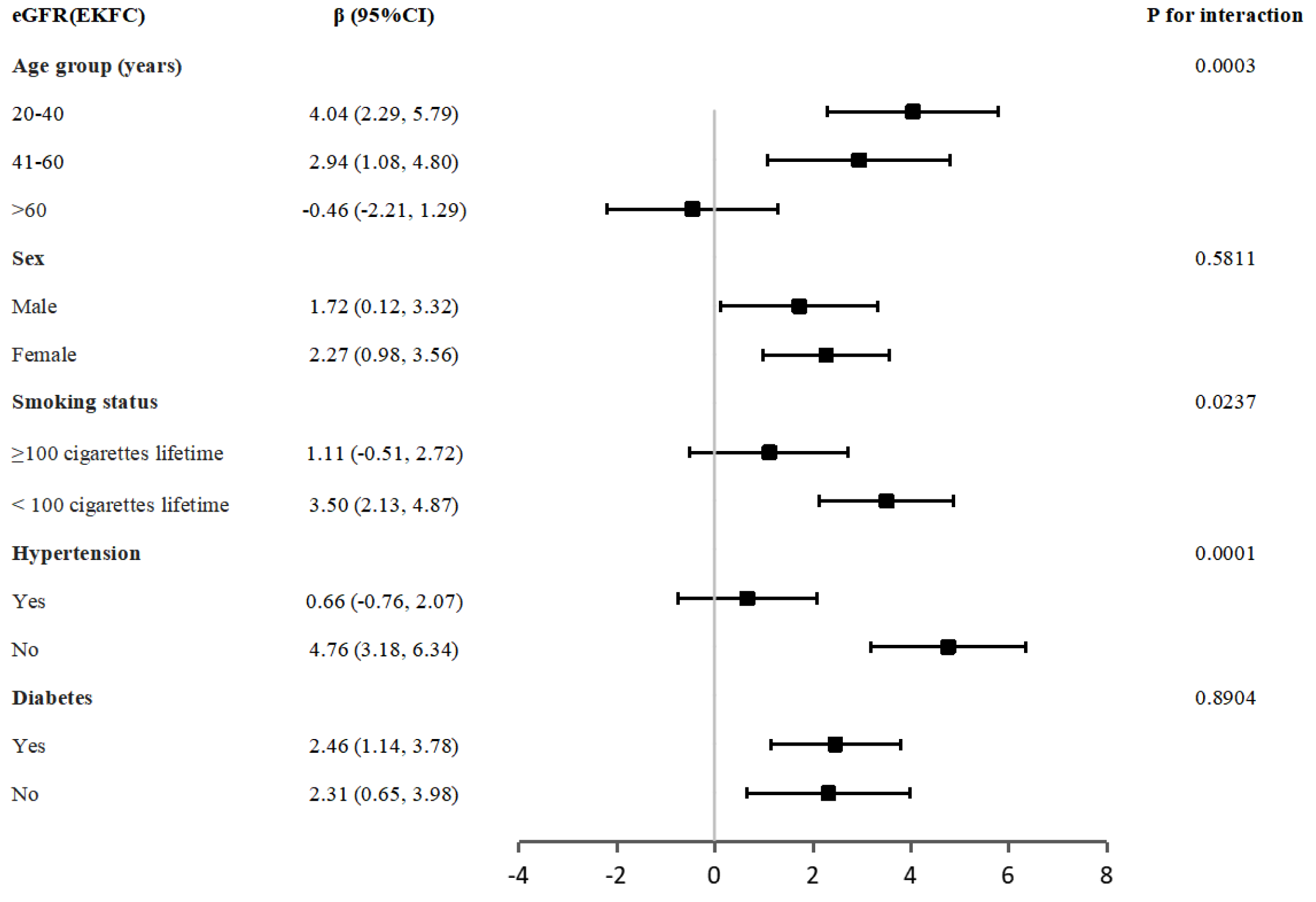

Supplement: Supplementary file 7 — Additional file 7. Supplementary Figure S7. Subgroup analysis for the association of WWI and eGFR(EKFC). [file 12882_2023_3316_MOESM7_ESM.tif]
