# Supplementary material for: Association between weight-adjusted-waist index and chronic kidney disease: a cross-sectional study
Source: BMC Nephrol. 2023 Sep 11;24:266. doi: 10.1186/s12882-023-03316-w (PMC10494374; doi:10.1186/s12882-023-03316-w)
Supplement: Supplementary file 8 — Additional file 8. Supplementary Table S1. Associations between WWI and other obesity indicators with CKD (EKFC) and low-eGFR (EKFC). [file 12882_2023_3316_MOESM8_ESM.docx]

**Supplementary Table S1 |** Association between WWI and other obesity indicators with CKD (EKFC) and low-eGFR (EKFC).

| **Index** | **Outcome** | **Continuous or categories** | Model 1^3^ | | Model 2^4^ | | [Model 3](https://www.ncbi.nlm.nih.gov/pmc/articles/PMC8987107/table/T2/?report=objectonly#t2fna)^5^ | |
| --- | --- | --- | --- | --- | --- | --- | --- | --- |
|  |  |  | OR^1^ (95%CI^2^) | *P-* value | OR (95%CI) | *P-* value | OR (95%CI) | *P-* value |
| **WWI** | **CKD(EKFC)** | WWI as continuous variable | 1.27 (1.24, 1.31) | <0.0001 | 1.31 (1.27, 1.34) | <0.0001 | 1.40 (1.24, 1.58) | <0.0001 |
|  |  | Tertile 1 | Reference |  | Reference |  | Reference |  |
|  |  | Tertile 2 | 0.93 (0.88, 1.00) | 0.0388 | 0.97 (0.91, 1.04) | 0.4513 | 0.96 (0.73, 1.28) | 0.7985 |
|  |  | Tertile 3 | 1.65 (1.56, 1.75) | <0.0001 | 1.77 (1.66, 1.88) | <0.0001 | 1.80 (1.37, 2.36) | <0.0001 |
|  |  | *P* for trend | <0.0001 |  | <0.0001 |  | <0.0001 |  |
|  | **Low-eGFR(EKFC)** | WWI as continuous variable | 1.27 (1.23, 1.32) | <0.0001 | 1.34 (1.29, 1.39) | <0.0001 | 1.08 (0.93, 1.24) | 0.3229 |
|  |  | Tertile 1 | Reference |  | Reference |  | Reference |  |
|  |  | Tertile 2 | 1.05 (0.96, 1.14) | 0.2810 | 1.15 (1.05, 1.26) | 0.0028 | 0.67 (0.47, 0.97) | 0.0325 |
|  |  | Tertile 3 | 1.74 (1.60, 1.88) | <0.0001 | 2.01 (1.85, 2.18) | <0.0001 | 1.08 (0.77, 1.52) | 0.6696 |
|  |  | *P* for trend | <0.0001 |  | <0.0001 |  | 0.2383 |  |
| **BMI** | **CKD(EKFC)** | BMI as continuous variable | 1.01 (1.01, 1.01) | <0.0001 | 1.01 (1.01, 1.02) | <0.0001 | 1.01 (0.99, 1.02) | 0.4130 |
|  |  | Tertile 1 | Reference |  | Reference |  | Reference |  |
|  |  | Tertile 2 | 1.08 (1.02, 1.15) | 0.0109 | 1.14 (1.07, 1.22) | <0.0001 | 0.84 (0.65, 1.10) | 0.2133 |
|  |  | Tertile 3 | 1.24 (1.17, 1.32) | <0.0001 | 1.34 (1.25, 1.42) | <0.0001 | 1.15 (0.88, 1.51) | 0.3098 |
|  |  | *P* for trend | <0.0001 |  | <0.0001 |  | 0.0971 |  |
|  | **Low-eGFR(EKFC)** | BMI as continuous variable | 1.03 (1.02, 1.03) | <0.0001 | 1.04 (1.03, 1.04) | <0.0001 | 1.00 (0.98, 1.02) | 0.9003 |
|  |  | Tertile 1 | Reference |  | Reference |  | Reference |  |
|  |  | Tertile 2 | 1.83 (1.68, 1.99) | <0.0001 | 2.14 (1.96, 2.34) | <0.0001 | 1.47 (1.04, 2.07) | 0.0298 |
|  |  | Tertile 3 | 1.84 (1.69, 2.00) | <0.0001 | 2.23 (2.04, 2.44) | <0.0001 | 1.28 (0.90, 1.82) | 0.1617 |
|  |  | *P* for trend | <0.0001 |  | <0.0001 |  | 0.3978 |  |
| **WHTR** | **CKD(EKFC)** | WHTR as continuous variable | 4.38 (3.45, 5.56) | <0.0001 | 6.04 (4.72, 7.72) | <0.0001 | 5.92 (2.02, 17.34) | 0.0012 |
|  |  | Tertile 1 | Reference |  | Reference |  | Reference |  |
|  |  | Tertile 2 | 1.04 (0.97, 1.10) | 0.2685 | 1.09 (1.02, 1.16) | 0.0105 | 0.98 (0.74, 1.29) | 0.8932 |
|  |  | Tertile 3 | 1.48 (1.39, 1.57) | <0.0001 | 1.60 (1.50, 1.70) | <0.0001 | 1.61 (1.21, 2.13) | 0.0010 |
|  |  | *P* for trend | <0.0001 |  | <0.0001 |  | <0.0001 |  |
|  | **Low-eGFR(EKFC)** | WHTR as continuous variable | 8.98 (6.62, 12.17) | <0.0001 | 29.18 (20.43, 41.66) | <0.0001 | 2.31 (0.58, 9.27) | 0.2358 |
|  |  | Tertile 1 | Reference |  | Reference |  | Reference |  |
|  |  | Tertile 2 | 1.59 (1.46, 1.73) | <0.0001 | 1.83 (1.67, 2.00) | <0.0001 | 1.09 (0.76, 1.56) | 0.6247 |
|  |  | Tertile 3 | 1.87 (1.72, 2.04) | <0.0001 | 2.26 (2.07, 2.47) | <0.0001 | 1.13 (0.79, 1.62) | 0.4972 |
|  |  | *P* for trend | <0.0001 |  | <0.0001 |  | 0.5181 |  |
| **Height** | **CKD(EKFC)** | Height as continuous variable | 1.02 (1.01, 1.02) | <0.0001 | 1.02 (1.02, 1.02) | <0.0001 | 0.99 (0.99, 1.00) | 0.3189 |
|  |  | Tertile 1 | Reference |  | Reference |  | Reference |  |
|  |  | Tertile 2 | 1.08 (1.01, 1.15) | 0.0177 | 1.12 (1.05, 1.19) | 0.0007 | 0.91 (0.71, 1.17) | 0.4747 |
|  |  | Tertile 3 | 1.46 (1.37, 1.55) | <0.0001 | 1.58 (1.48, 1.68) | <0.0001 | 0.88 (0.69, 1.12) | 0.2970 |
|  |  | *P* for trend | <0.0001 |  | <0.0001 |  | 0.3072 |  |
|  | **Low-eGFR(EKFC)** | Height as continuous variable | 1.05 (1.05, 1.05) | <0.0001 | 1.06 (1.06, 1.07) | <0.0001 | 1.05 (1.04, 1.07) | <0.0001 |
|  |  | Tertile 1 | Reference |  | Reference |  | Reference |  |
|  |  | Tertile 2 | 1.63 (1.48, 1.79) | <0.0001 | 1.83 (1.66, 2.03) | <0.0001 | 2.01 (1.42, 2.85) | <0.0001 |
|  |  | Tertile 3 | 3.33 (3.05, 3.64) | <0.0001 | 4.45 (4.05, 4.89) | <0.0001 | 3.31 (2.37, 4.62) | <0.0001 |
|  |  | *P* for trend | <0.0001 |  | <0.0001<0.0001 |  | <0.0001 |  |
| **Weight** | **CKD(EKFC)** | Weight as continuous variable | 1.01 (1.00, 1.01) | <0.0001 | 1.01 (1.01, 1.01) | <0.0001 | 1.00 (0.99, 1.01) | 0.6265 |
|  |  | Tertile 1 | Reference |  | Reference |  | Reference |  |
|  |  | Tertile 2 | 1.12 (1.05, 1.19) | 0.0005 | 1.18 (1.10, 1.26) | <0.0001 | 0.82 (0.63, 1.07) | 0.1431 |
|  |  | Tertile 3 | 1.40 (1.32, 1.49) | <0.0001 | 1.54 (1.45, 1.64) | <0.0001 | 0.92 (0.71, 1.19) | 0.5286 |
|  |  | *P* for trend | <0.0001 |  | <0.0001 |  | 0.7708 |  |
|  | **Low-eGFR(EKFC)** | Weight as continuous variable | 1.02 (1.01, 1.02) | <0.0001 | 1.02 (1.02, 1.02) | <0.0001 | 1.01 (1.01, 1.02) | <0.0001 |
|  |  | Tertile 1 | Reference |  | Reference |  | Reference |  |
|  |  | Tertile 2 | 2.32 (2.11, 2.55) | <0.0001 | 2.78 (2.51, 3.06) | <0.0001 | 1.89 (1.31, 2.72) | 0.0006 |
|  |  | Tertile 3 | 3.07 (2.81, 3.37) | <0.0001 | 4.13 (3.75, 4.55) | <0.0001 | 2.19 (1.53, 3.14) | <0.0001 |
|  |  | *P* for trend | <0.0001 |  | <0.0001 |  | <0.0001 |  |
| **WC** | **CKD(EKFC)** | WC as continuous variable | 1.01 (1.01, 1.01) | <0.0001 | 1.01 (1.01, 1.02) | <0.0001 | 1.01 (1.00, 1.02) | 0.0025 |
|  |  | Tertile 1 | Reference |  | Reference |  | Reference |  |
|  |  | Tertile 2 | 1.08 (1.02, 1.15) | 0.0149 | 1.15 (1.08, 1.23) | <0.0001 | 0.91 (0.69, 1.21) | 0.5203 |
|  |  | Tertile 3 | 1.62 (1.52, 1.72) | <0.0001 | 1.80 (1.69, 1.92) | <0.0001 | 1.36 (1.02, 1.80) | 0.0369 |
|  |  | *P* for trend | <0.0001 |  | <0.0001 |  | 0.0058 |  |
|  | **Low-eGFR(EKFC)** | WC as continuous variable | 1.02 (1.02, 1.02) | <0.0001 | 1.03 (1.03, 1.03) | <0.0001 | 1.02 (1.01, 1.03) | 0.0002 |
|  |  | Tertile 1 | Reference |  | Reference |  | Reference |  |
|  |  | Tertile 2 | 1.97 (1.80, 2.16) | <0.0001 | 2.37 (2.15, 2.61) | <0.0001 | 1.77 (1.20, 2.63) | 0.0043 |
|  |  | Tertile 3 | 2.79 (2.55, 3.04) | <0.0001 | 3.69 (3.36, 4.05) | <0.0001 | 2.10 (1.42, 3.11) | 0.0002 |
|  |  | *P* for trend | <0.0001 |  | <0.0001 |  | 0.0005 |  |

In sensitivity analysis, WWI, BMI, WHTR, WC, height and weight were converted from continuous variables to categorical variables (tertiles).

^1^OR: Odd ratio.

^2^95% CI: 95% confidence interval.

^3^Model 1: No covariates were adjusted.

^4^Model 2: Adjusted for age, sex, and race.

^5^Model 3: Adjusted for sex, age, race, education level, smoking status, serum uric acid, TC, LDL-C, HDL-C, triglycerides, serum total calcium, hypertension, and diabetes status.
