# Supplementary material for: Association between weight-adjusted-waist index and chronic kidney disease: a cross-sectional study
Source: BMC Nephrol. 2023 Sep 11;24:266. doi: 10.1186/s12882-023-03316-w (PMC10494374; doi:10.1186/s12882-023-03316-w)
Supplement: Supplementary file 9 — Additional file 9. Supplementary Table S2. Threshold effect analysis of WWI and other obesity indicators on CKD(EKFC) and low-eGFR(EKFC) using a two-piecewise linear regression model in Model 3. [file 12882_2023_3316_MOESM9_ESM.docx]

**Supplementary Table S2 |** Threshold effect analysis of WWI and other obesity indicators on CKD(EKFC) and low-eGFR(EKFC) using a two-piecewise linear regression model in Model 3.

|  | CKD(EKFC) | | Low-eGFR(EKFC) | |
| --- | --- | --- | --- | --- |
|  | OR^1^ (95%CI^2^) | *P-* value | OR (95%CI) | *P-* value |
| **WWI** |  |  |  |  |
| **Fitting by standard linear model** | 1.40 (1.24, 1.58) | <0.0001 | 1.08 (0.93, 1.24) | 0.3229 |
| **Fitting by two-piecewise linear model** |  |  |  |  |
| Breakpoint (K) | 10.44 |  | 9.58 |  |
| OR1(< K ) | 0.88 (0.61, 1.27) | 0.4873 | 0.19 (0.05, 0.66) | 0.0093 |
| OR2(> K ) | 1.62 (1.37, 1.90) | <0.0001 | 1.17 (1.00, 1.37) | 0.0519 |
| OR2 / OR1 | 1.84 (1.16, 2.91) | 0.0090 | 6.26 (1.67, 23.49) | 0.0066 |
| Logarithmic likelihood ratio test P-value | 0.009 |  | 0.009 |  |
| **BMI** |  |  |  |  |
| **Fitting by standard linear model** | 1.01 (0.99, 1.02) | 0.4130 | 1.00 (0.98, 1.02) | 0.9003 |
| **Fitting by two-piecewise linear model** |  |  |  |  |
| Breakpoint (K) | 19.9 |  | 26.4 |  |
| OR1(< K ) | 0.68 (0.53, 0.89) | 0.0042 | 1.09 (1.02, 1.17) | 0.0126 |
| OR2(> K ) | 1.01 (0.99, 1.03) | 0.1144 | 0.98 (0.96, 1.01) | 0.1209 |
| OR2 / OR1 | 1.48 (1.14, 1.93) | 0.0036 | 0.90 (0.83, 0.97) | 0.0099 |
| Logarithmic likelihood ratio test P-value | 0.003 |  | 0.009 |  |
| **WHTR** |  |  |  |  |
| **Fitting by standard linear model** | 5.92 (2.02, 17.34) | 0.0012 | 1.52 (0.40, 5.78) | 0.5382 |
| **Fitting by two-piecewise linear model** |  |  |  |  |
| Breakpoint (K) | 0.5 |  | 0.44 |  |
| OR1(< K ) | 0.01 (0.01, 2.78) | 0.1094 | 3.50 (0.01, 4.34) | 0.1291 |
| OR2(> K ) | 12.67 (3.57, 44.94) | <0.0001 | 1.11 (0.28, 4.46) | 0.8860 |
| OR2 / OR1 | 12.62 (2.59, 47.89) | 0.0236 | 0.01 (0.01,98.68) | 0.1336 |
| Logarithmic likelihood ratio test P-value | 0.024 |  | 0.092 |  |
| **Height** |  |  |  |  |
| **Fitting by standard linear model** | 1.00 (0.99, 1.00) | 0.3189 | 1.05 (1.04, 1.07) | <0.0001 |
| **Fitting by two-piecewise linear model** |  |  |  |  |
| Breakpoint (K) | 151 |  | 169.7 |  |
| OR1(< K ) | 0.95 (0.86, 1.04) | 0.2675 | 1.08 (1.05, 1.10) | <0.0001 |
| OR2(> K ) | 1.00 (0.99, 1.01) | 0.5778 | 1.03 (1.00, 1.06) | 0.0233 |
| OR2 / OR1 | 1.05 (0.95, 1.17) | 0.3103 | 0.96 (0.92, 0.99) | 0.0499 |
| Logarithmic likelihood ratio test P-value | 0.312 |  | 0.048 |  |
| **Weight** |  |  |  |  |
| **Fitting by standard linear model** | 1.00 (0.99, 1.01) | 0.6265 | 1.01 (1.01, 1.02) | <0.0001 |
| **Fitting by two-piecewise linear model** |  |  |  |  |
| Breakpoint (K) | 67.8 |  | 79 |  |
| OR1(< K ) | 0.98 (0.96, 0.99) | 0.0304 | 1.04 (1.02, 1.06) | <0.0001 |
| OR2(> K ) | 1.01 (0.99, 1.01) | 0.0768 | 1.00 (0.99, 1.01) | 0.6523 |
| OR2 / OR1 | 1.03 (1.00, 1.05) | 0.0186 | 0.96 (0.94, 0.99) | 0.0014 |
| Logarithmic likelihood ratio test P-value | 0.019 |  | 0.001 |  |
| **WC** |  |  |  |  |
| **Fitting by standard linear model** | 1.01 (1.00, 1.02) | 0.0025 | 1.02 (1.01, 1.02) | 0.0002 |
| **Fitting by two-piecewise linear model** |  |  |  |  |
| Breakpoint (K) | 79.1 |  | 74.4 |  |
| OR1(< K ) | 0.95 (0.90, 0.99) | 0.0402 | 1.63 (1.04, 2.56) | 0.0344 |
| OR2(> K ) | 1.01 (1.01, 1.02) | 0.0002 | 1.01 (1.00, 1.02) | 0.0041 |
| OR2 / OR1 | 1.07 (1.01, 1.13) | 0.0147 | 0.62 (0.40, 0.98) | 0.0399 |
| Logarithmic likelihood ratio test P-value | 0.015 |  | 0.004 |  |

Adjusted for sex, age, race, education level, smoking status, serum uric acid, TC, LDL-C, HDL-C, triglycerides, serum total calcium, hypertension, and diabetes status.

^1^OR: Odd ratio.

^2^95% CI: 95% confidence interval.
