# Supplementary material for: Association between weight-adjusted-waist index and chronic kidney disease: a cross-sectional study
Source: BMC Nephrol. 2023 Sep 11;24:266. doi: 10.1186/s12882-023-03316-w (PMC10494374; doi:10.1186/s12882-023-03316-w)
Supplement: Supplementary file 10 — Additional file 10. Supplementary Table S3. Comparison of AUC values between WWI and other obesity indicators. [file 12882_2023_3316_MOESM10_ESM.docx]

**Supplementary Table S3 |** Comparison of AUC values between WWI and other obesity indicators.

| Test | AUC^1^ | 95%CI^2^ low | 95%CI upp | Best threshold | Specificity | Sensitivity | *P* for different in AUC |
| --- | --- | --- | --- | --- | --- | --- | --- |
| **CKD(EKFC)** |  |  |  |  |  |  |  |
| WWI | 0.5653 | 0.5578 | 0.5728 | 11.2105 | 0.6854 | 0.4405 | Reference |
| BMI | 0.5228 | 0.5155 | 0.53 | 26.995 | 0.5403 | 0.5035 | <0.0001 |
| WHTR | 0.5457 | 0.5383 | 0.5531 | 0.588 | 0.6254 | 0.4665 | <0.0001 |
| Height | 0.5463 | 0.5391 | 0.5536 | 167.75 | 0.5859 | 0.5014 | 0.0022 |
| Weight | 0.5400 | 0.5328 | 0.5473 | 72.27 | 0.4848 | 0.5841 | <0.0001 |
| WC | 0.5596 | 0.5523 | 0.567 | 94.55 | 0.5569 | 0.5515 | 0.0366 |
| **Low-eGFR(EKFC)** |  |  |  |  |  |  |  |
| WWI | 0.5686 | 0.5589 | 0.5784 | 11.1505 | 0.6523 | 0.4773 | <0.0001 |
| BMI | 0.5688 | 0.5604 | 0.5773 | 23.455 | 0.3098 | 0.8137 | <0.0001 |
| WHTR | 0.5746 | 0.5658 | 0.5835 | 0.5429 | 0.4552 | 0.6732 | <0.0001 |
| Height | 0.6452 | 0.6364 | 0.654 | 167.75 | 0.5937 | 0.6472 | Reference |
| Weight | 0.6241 | 0.6158 | 0.6323 | 71.55 | 0.4766 | 0.7178 | <0.0001 |
| WC | 0.6193 | 0.6109 | 0.6277 | 92.65 | 0.5122 | 0.6716 | <0.0001 |

^1^AUC: area under the curve.

^2^95% CI: 95% confidence interval.
