# Supplementary material for: Association between weight-adjusted-waist index and chronic kidney disease: a cross-sectional study
Source: BMC Nephrol. 2023 Sep 11;24:266. doi: 10.1186/s12882-023-03316-w (PMC10494374; doi:10.1186/s12882-023-03316-w)
Supplement: Supplementary file 11 — Additional file 11. Supplementary Table S4. Association between WWI and eGFR. [file 12882_2023_3316_MOESM11_ESM.docx]

**Supplementary Table S4 |** Association between WWI and eGFR.

| **Index** | **Outcome** | **Continuous or categories** | Model 1^3^ | | Model 2^4^ | | [Model 4](https://www.ncbi.nlm.nih.gov/pmc/articles/PMC8987107/table/T2/?report=objectonly#t2fna)^5^ | |
| --- | --- | --- | --- | --- | --- | --- | --- | --- |
|  |  |  | β^1^ (95%CI^2^) | *P-* value | β (95%CI) | *P-* value | β (95%CI) | *P-* value |
| **WWI** | **CKD^6^** | WWI as continuous variable | 0.37 (0.07, 0.67) | 0.0158 | 0.03 (-0.23, 0.29) | 0.8414 | 4.61 (3.04, 6.19) | <0.0001 |
|  |  | Tertile 1 | Reference |  | Reference |  | Reference |  |
|  |  | Tertile 2 | 1.77 (1.09, 2.46) | <0.0001 | 0.72 (0.13, 1.31) | 0.0173 | 7.08 (3.40, 10.76) | 0.0002 |
|  |  | Tertile 3 | -0.34 (-1.02, 0.35) | 0.3372 | -1.21 (-1.80, -0.62) | <0.0001 | 10.24 (6.59, 13.88) | <0.0001 |
|  |  | *P* for trend | 0.3491 |  | <0.0001 |  | <0.0001 |  |

In sensitivity analysis, WWI was converted from a continuous variable to a categorical variable (tertiles).

^1^β: effect size.

^2^95% CI: 95% confidence interval.

^3^Model 1: No covariates were adjusted.

^4^Model 2: Adjusted for age, sex, and race.

^5^Model 4: Adjusted for albuminuria, sex, age, race, education level, smoking status, serum uric acid, TC, LDL-C, HDL-C, triglycerides, serum total calcium, hypertension, and diabetes status.
