# Supplementary material for: Association between weight-adjusted-waist index and chronic kidney disease: a cross-sectional study
Source: BMC Nephrol. 2023 Sep 11;24:266. doi: 10.1186/s12882-023-03316-w (PMC10494374; doi:10.1186/s12882-023-03316-w)
Supplement: Supplementary file 12 — Additional file 12. Supplementary Table S5. Threshold effect analysis of WWI on eGFR using a two-piecewise linear regression model in Model 1 and Model 4. [file 12882_2023_3316_MOESM12_ESM.docx]

**Supplementary Table S5 |** Threshold effect analysis of WWI on eGFR using a two-piecewise linear regression model in Model 1 and Model 4.

|  | Model 1^3^ | | Model 4^4^ | |
| --- | --- | --- | --- | --- |
| **WWI** | β^1^ (95%CI^2^) | *P-* value | β (95%CI) | *P-* value |
| **Fitting by standard linear model** | 0.37 (0.07, 0.67) | 0.0158 | 4.61 (3.04, 6.19) | <0.0001 |
| **Fitting by two-piecewise linear model** |  |  |  |  |
| Breakpoint (K) | 9.9 |  | 10.62 |  |
| OR1(< K ) | 12.24 (10.79, 13.70) | <0.0001 | 10.40 (6.29, 14.52) | <0.0001 |
| OR2(> K ) | -1.55 (-1.93, -1.17) | <0.0001 | 2.12 (-0.14, 4.39) | 0.0663 |
| OR2 / OR1 | -13.79 (-15.45, -12.14) | <0.0001 | -8.28 (-13.71, -2.85) | 0.0029 |
| Logarithmic likelihood ratio test P-value | <0.0001 |  | 0.003 |  |

^1^β: effect size.

^2^95% CI: 95% confidence interval.

^3^Model 1: No covariates were adjusted.

^4^Model 4: Adjusted for albuminuria, sex, age, race, education level, smoking status, serum uric acid, TC, LDL-C, HDL-C, triglycerides, serum total calcium, hypertension, and diabetes status.
