# Supplementary material for: Association between weight-adjusted-waist index and chronic kidney disease: a cross-sectional study
Source: BMC Nephrol. 2023 Sep 11;24:266. doi: 10.1186/s12882-023-03316-w (PMC10494374; doi:10.1186/s12882-023-03316-w)
Supplement: Supplementary file 13 — Additional file 13. Supplementary Table S6. Association between WWI and eGFR (EKFC). [file 12882_2023_3316_MOESM13_ESM.docx]

**Supplementary Table S6 |** Association between WWI and eGFR (EKFC).

| **Index** | **Outcome** | **Continuous or categories** | Model 1^3^ | | Model 2^4^ | | [Model 4](https://www.ncbi.nlm.nih.gov/pmc/articles/PMC8987107/table/T2/?report=objectonly#t2fna)^5^ | |
| --- | --- | --- | --- | --- | --- | --- | --- | --- |
|  |  |  | β^1^ (95%CI^2^) | *P-* value | β (95%CI) | *P-* value | β (95%CI) | *P-* value |
| **WWI** | **CKD(EKFC)** | WWI as continuous variable | 0.04 (-0.19, 0.26) | 0.7629 | -0.34 (-0.52, -0.15) | 0.0004 | 2.42 (1.36, 3.48) | <0.0001 |
|  |  | Tertile 1 | Reference |  | Reference |  | Reference |  |
|  |  | Tertile 2 | 1.49 (0.97, 2.01) | <0.0001 | 0.43 (0.01, 0.85) | 0.0467 | 4.83 (2.36, 7.30) | 0.0001 |
|  |  | Tertile 3 | -0.69 (-1.20, -0.17) | 0.0094 | -1.61 (-2.03, -1.19) | <0.0001 | 5.54 (3.09, 7.98) | <0.0001 |
|  |  | *P* for trend | 0.0103 |  | <0.0001 |  | <0.0001 |  |

In sensitivity analysis, WWI was converted from a continuous variable to a categorical variable (tertiles).

^1^β: effect size.

^2^95% CI: 95% confidence interval.

^3^Model 1: No covariates were adjusted.

^4^Model 2: Adjusted for age, sex, and race.

^5^Model 4: Adjusted for albuminuria, sex, age, race, education level, smoking status, serum uric acid, TC, LDL-C, HDL-C, triglycerides, serum total calcium, hypertension, and diabetes status.
