# Supplementary material for: Association between weight-adjusted-waist index and chronic kidney disease: a cross-sectional study
Source: BMC Nephrol. 2023 Sep 11;24:266. doi: 10.1186/s12882-023-03316-w (PMC10494374; doi:10.1186/s12882-023-03316-w)
Supplement: Supplementary file 14 — Additional file 14. Supplementary Table S7. Threshold effect analysis of WWI on eGFR (EKFC) using a two-piecewise linear regression model in Model 1 and Model 4. [file 12882_2023_3316_MOESM14_ESM.docx]

**Supplementary Table S7 |** Threshold effect analysis of WWI on eGFR (EKFC) using a two-piecewise linear regression model in Model 1 and Model 4.

|  | Model 1^3^ | | Model 4^4^ | |
| --- | --- | --- | --- | --- |
| **WWI** | β^1^ (95%CI^2^) | *P-* value | β (95%CI) | *P-* value |
| **Fitting by standard linear model** | 0.04 (-0.19, 0.26) | 0.7629 | 2.42 (1.36, 3.48) | <0.0001 |
| **Fitting by two-piecewise linear model** |  |  |  |  |
| Breakpoint (K) | 10.02 |  | 10.62 |  |
| OR1(< K ) | 8.08 (7.13, 9.03) | <0.001 | 6.24 (3.48, 9.00) | <0.0001 |
| OR2(> K ) | -1.67 (-1.97, -1.37) | <0.0001 | 0.78 (-0.74, 2.30) | 0.3156 |
| OR2 / OR1 | -9.75 (-10.87, -8.63) | <0.001 | -5.47 (-9.11, -1.82) | 0.0033 |
| Logarithmic likelihood ratio test P-value | <0.001 |  | 0.003 |  |

^1^β: effect size.

^2^95% CI: 95% confidence interval.

^3^Model 1: No covariates were adjusted.

^4^Model 4: Adjusted for albuminuria, sex, age, race, education level, smoking status, serum uric acid, TC, LDL-C, HDL-C, triglycerides, serum total calcium, hypertension, and diabetes status.
